# Microbial degradation of contaminants of emerging concern: metabolic, genetic and omics insights for enhanced bioremediation

**DOI:** 10.3389/fbioe.2024.1470522

**Published:** 2024-09-19

**Authors:** Bhavik A. Shah, Harshit Malhotra, Sandesh E. Papade, Tushar Dhamale, Omkar P. Ingale, Sravanti T. Kasarlawar, Prashant S. Phale

**Affiliations:** Department of Biosciences and Bioengineering, Indian Institute of Technology-Bombay, Mumbai, India

**Keywords:** biodegradation, pharmaceuticals, plasticizers, cyanotoxins, pesticides, omics, metabolic pathways, metabolic engineering

## Abstract

The perpetual release of natural/synthetic pollutants into the environment poses major risks to ecological balance and human health. Amongst these, contaminants of emerging concern (CECs) are characterized by their recent introduction/detection in various niches, thereby causing significant hazards and necessitating their removal. Pharmaceuticals, plasticizers, cyanotoxins and emerging pesticides are major groups of CECs that are highly toxic and found to occur in various compartments of the biosphere. The sources of these compounds can be multipartite including industrial discharge, improper disposal, excretion of unmetabolized residues, eutrophication *etc*., while their fate and persistence are determined by factors such as physico-chemical properties, environmental conditions, biodegradability and hydrological factors. The resultant exposure of these compounds to microbiota has imposed a selection pressure and resulted in evolution of metabolic pathways for their biotransformation and/or utilization as sole source of carbon and energy. Such microbial degradation phenotype can be exploited to clean-up CECs from the environment, offering a cost-effective and eco-friendly alternative to abiotic methods of removal, thereby mitigating their toxicity. However, efficient bioprocess development for bioremediation strategies requires extensive understanding of individual components such as pathway gene clusters, proteins/enzymes, metabolites and associated regulatory mechanisms. “Omics” and “Meta-omics” techniques aid in providing crucial insights into the complex interactions and functions of these components as well as microbial community, enabling more effective and targeted bioremediation. Aside from natural isolates, metabolic engineering approaches employ the application of genetic engineering to enhance metabolic diversity and degradation rates. The integration of omics data will further aid in developing systemic-level bioremediation and metabolic engineering strategies, thereby optimising the clean-up process. This review describes bacterial catabolic pathways, genetics, and application of omics and metabolic engineering for bioremediation of four major groups of CECs: pharmaceuticals, plasticizers, cyanotoxins, and emerging pesticides.

## 1 Introduction

The human population is perpetually interacting with a wide range of external chemicals, including both man-made and naturally occurring compounds. The impact of this continuous exposure can be either beneficial or detrimental to human health. While certain compounds such as pharmaceuticals, pesticides, plasticizers *etc.,* have contributed immensely to development and sustenance (Table 1), their excessive usage has led to distribution and persistence in various ecosystems, causing disruption and toxic effects. Amongst these, “contaminants of emerging concern” (CEC) are naturally occurring or synthetic compounds which are recently detected/suspected to be present in various habitats and might significantly impact the metabolism of living organisms. The detection of such compounds can be attributed either to their recent introduction into the environment or an advancement in detection technologies. Additionally, CECs also include known contaminants with developing or poorly understood risk profiles (Sauvé and Desrosiers, 2014). Examples of CECs include compounds such as pharmaceuticals, personal care products, nanomaterials, pesticides, plasticizers, microplastics, radionuclides/rare earth elements, cyano/algal toxins and perfluorinated compounds.

**TABLE 1:** List of contaminants of emerging concern detailing characteristics, toxic effects, applications, and microbial genus involved in degradation.

Compound (Pubchem ID)	Organisms involved in degradation	Application	Health effects in humans	LD_50_ values (Rat oral)	Permissible limits in drinking water	Global usage data in metric tonnes	References
*Antibiotics*							
Sulfamethazine (5327)	*Achromobacter,* *Arthrobacter,* *Bacillus,* *Geobacillus*, *Microbacterium* *,*	Treatment of urinary tract infections, pneumonia, *chlamydia*, bronchitis, ear infections in humans and livestock	Hypersensitivity, hepatotoxicity, nephrotoxicity, hematological effects, neurotoxicity, gastrointenstinal effects	7,000 mg/kg	-	>15,000 tons	Weidner-Wells and Macielag, 2003, Huang et al., 2012, Baran et al., 2012, Pan et al., 2017, Cao et al., 2019, Billet et al., 2021, Ovung and Bhattacharyya, 2021, Dong et al., 2022
Sulfamethoxazole (5329)	*Achromobacter*, *Microbacterium*, *Paenarthrobacter*, *Pseudomonas*			6,200 mg/kg	-		Reis et al., 2014, Jiang et al., 2014, Ricken et al., 2015, Wang and Wang, 2018, Qi et al., 2021
Sulphadiazine (5215)	*Alcaligenes*, *Arthrobacter*, *Bacillus*, *Microbacterium, Pimelobacter*			1,500 mg/kg	-		Tappe et al., 2013, Deng et al., 2016, Deng et al., 2018, Chen et al., 2019, Du et al., 2023
Sulphamethoxy diazine (5326)	*Alcaligenes*, *Arthrobacter*			6,000 mg/kg	-		Deng et al., 2016, Du et al., 2023
Penicillin (5904)	*Burkholderia*, *Pandorea*, *Pseudomonas*, *Sphingobacterium*	Treatment of pneumonia, syphilis, meningitis, strep throat in humans and livestock	Nausea, vomiting, diarrhea, skin rashes, neurotoxicity including seizures	6,600 mg/kg	-	**-**	Miller, 2002, Crofts et al., 2018, Zhang et al., 2024
Erythromycin (12560)	*Ochrobactrum*, *Paracoccus*, *Pseudomonas, Rhodococcus*	Treatment of bacterial respiratory tract infections, treatment of *Pertussis*	Immunostimulation, cardiotoxicity, allergic reactions, oxidative stress, genotoxicity, hypersensitivity	4,600 mg/kg	-	**-**	Berthet et al., 2010, Mao et al., 2013, Zhang et al., 2017c, Ren et al., 2022, Ren et al., 2023a
Chloramphenicol (5959)	*Aeromonas*, *Burkholderia*, Sphingobium, Sphingomonas	Treatment of *Salmonella* infections, meningitis, Rickettsial infections, topical applications, anaerobic infections	Hematological effects, gray baby syndrome, neurotoxicity, gastrointenstinal effects	2,500 mg/kg	-	**-**	Feder et al., 1981, Eliakim-Raz et al., 2015, Zhang et al., 2020b, Tan et al., 2022a, Gao et al., 2024a
Ciprofloxacin (2764)	*Archromobacter*, *Bacillus*, *Enterococcus*, Lactococcus, *Ochrobactrum*	Treatment of urinary tract infections, respiratory tract, skin, soft tissue, bones and gut	Neurotoxicity, hepatoxicity, nephrotoxicity, musculoskeletal effects	1,280 mg/kg	-	**-**	Ball, 1986, Badawy et al., 2021, Feng et al., 2019
*Analgesics*							
Ibuprofen (3672)	*Bacillus*, *Micrococcus*, *Novosphingobium,* *Rhizorhabdus*, *Sphingomonas*, *Sphingopyxis*, *Variovorax*	Used as analgesic, anti-inflammatory, antipyretic, cardioprotective	Hepatotoxicity, nephrotoxicity, neurotoxicity, gastrointestinal effects	636 mg/kg	-	**-**	Murdoch and Hay, 2013, Murdoch and Hay, 2015, Marchlewicz et al., 2017, Sharma et al., 2019, Rutere et al., 2020, Aguilar-Romero et al., 2021, Aulestia et al., 2022, Calisici et al., 2023
Acetaminophen (1983)	*Bacillus*, *Paracoccus*, *Pseudomonas*, *Rhodococcus*	Used as analgesic and antipyretic drug	Hepatotoxicity, nephrotoxicity, neurotoxicity	840 mg/kg	<71 ng/L	**-**	Ghanem et al., 2016, Vo et al., 2019, Chopra and Kumar, 2020, Akay and Tezel, 2020, Rios-Miguel et al., 2022, Pandey et al., 2024
Naproxen (156391)	*Bacillus*, *Planococcus,* *Pseudoxanthomonas* *Stenotrophomonas*	Used as anti-inflammatory agent and as analgesic to treat rheumatoid arthritis and other musculoskeletal disorders, dysmenorrhea, acute gout	Nephrotoxicity, neurotoxicity, gastrointestinal effects, cardiotoxicity	310 mg/kg	-	**-**	Wojcieszyńska et al., 2014, Domaradzka et al., 2015, Gorny et al., 2019, Lu et al., 2019, Wojcieszyńska and Guzik, 2020
*Steroid sex hormones*							
Testosterone (6013)	*Acinetobacter* *Comamonas*, *Novosphingobium,* *Pseudomonas,* *Sphingomonas*	Used in androgen replacement therapy, muscle wasting conditions, bone marrow failure syndrome	Testicular dysfunction, reproductive toxicity, neurotoxicity	**-**	-	**-**	Santer and Ajl, 1952, Oettel, 2003, Horinouchi et al., 2001, Roh and Chu, 2010, Yang et al., 2011, Ibero et al., 2019
Oestrogens (5757)	*Acinetobacter,* *Pseudomonas*, *Rhodococcus*, *Sphingomonas*	Used in hormone replacement therapy, contraception, treatment of certain cancers and osteoporosis	Reproductive toxicity, developmental toxicity, oxidative stress, inflammation	**-**	-	**-**	Adeel et al., 2017, Ke et al., 2007, Roh and Chu, 2010, Wang et al., 2019d, Harthern-Flint et al., 2021
*Miscellaneous*							
Fluoxetine (3386)	*Bacillus*, *Comamonas*, *Desulfomicrobium*, *Desulfovibrio*, *Pseudomonas*	Used as antidepressant to treat depression, panic disorder, bulimia, and obsessive-compulsive disorder	Hepatotoxicity, tachycardia, developmental toxicity	452 mg/kg	-	**-**	Stokes and Holtz, 1997, Khan and Murphy, 2021, Palma and Costa, 2021
Metformin (4091)	*Aminobacter*, *Microbacterium* *Pseudomonas,* *Sphingopyxis*	Used as antidiabetic to treat obesity, cancer, polycystic ovary syndrome and fatty liver disease	Lactic Acidosis, hepatotoxicity, nephrotoxicity, cardiotoxicity	1,000 mg/kg	-	-	Wang and Hoyte, 2018, Tassoulas et al., 2021, Robinson et al., 2021, Martinez-Vaz et al., 2022, Li et al., 2023
*Cyanotoxins*							
Microcystin-LR (445434)	*Novosphingobium*, *Sphingomonas*, *Sphingopyxis*, *Sphingosicicella*	Treatment of pulmonary fibrosis, organ, or tissue fibrosis	Hepatotoxicity, cytotoxicity, carcinogenicity, gastrointenstinal effects, nephrotoxicity neurotoxicity, suppresses phosphatase 2A activity altering the expression levels of miRNA, induces DNA damage, cytoskeleton disruption, autophagy and apoptosis	5 mg/kg	∼1 μg/L in drinking water	-	Hu et al., 2016a, Hu et al., 2016b, Bourne et al., 1996, Van Dolah, 2000, Maruyama et al., 2006, Žegura et al., 2011, Yan et al., 2012, Wang et al., 2017, Herrera et al., 2018, Svirčev et al., 2019, Wang et al., 2019e, WHO, 2020, Lee et al., 2021a, Lee et al., 2021b, WHO, 2021, Ren et al., 2024
Nodularin (4369034)	*Novosphingobium*, *Sphingomonas,* *Sphingopyxis*	**-**	Hepatotoxicity, gastrointestinal effects, respiratory toxicity, skin irritation, cytotoxicity, gastrointenstinal effects, nephrotoxicity neurotoxicity, inhibits phosphatases 1, 2A and 3 activity altering the expression levels of miRNA, inducing DNA damage, cytoskeleton disruption, autophagy and apoptosis	**-**	-	**-**	Feng et al., 2016, Chen et al., 2021c, Yuan et al., 2021
*Plasticizers*							
Di (2-ethylhexyl) phthalate (8343)	*Achromobacter,* *Acinetobacter,* *Agromyces,* *Bacillus,* *Burkholderia,* *Cupravidus,* *Gordonia,* *Microbacterium,* *Mycrobacterium,* *Pseudomonas,* *Rhodococcus*	Used as plasticizer in the production of plastics and PVC resins, PVA emulsion adhesives, etc	Endocrine disruption, reproductive and developmental toxicity, carcinogenicity, teratogenicity, endometriosis, nephrotoxicity, neurotoxicity, cardiotoxicity	>25 g/kg	>6–8 ppb in drinking water	6 billion tons	Koch et al., 2003, Zhao et al., 2016, Xu et al., 2017, Zhang et al., 2018, Fan et al., 2018, Kim et al., 2019b, Rowdhwal and Chen, 2018, Li et al., 2019, Wright et al., 2020, Chen et al., 2021a, Chen et al., 2021b, Kamaraj et al., 2022, Wang et al., 2021, Sun et al., 2024
Dibutyl Phthalate (3026)	*Acinetobacter,* *Arthrobacter,* *Bacillus,* *Cupravidus,* *Halomonas,* *Microbacterium*, *Mycobacterium,* *Pseudomonas*			8,000 mg/kg	<5 ppb in drinking water	220,000 tons	Feng et al., 2018, Wright et al., 2020, Czubacka et al., 2021, Feng et al., 2021, Chen et al., 2021a, Chen et al., 2021b, Nandi et al., 2021, Yan et al., 2021, Li et al., 2022, Jiang et al., 2022, Sun et al., 2024, Nahla et al., 2024
Benzyl butyl phthalate (2347)	*Acinetobacter, Arthrobacter,* *Bacillus,* *Gordonia*			2,330 mg/kg	-	**-**	Tyl et al., 2004, Ahmad et al., 2015, Roy et al., 2017, Zhang et al., 2018, Nandi et al., 2021, Kaur et al., 2021, Chatterjee and Dutta, 2003, Fan et al., 2023
Dioctyl phthalate (8346)	*Arthrobacter,* *Bacillus*, *Burkholderia,* *Gordonia,* *Rhodococcus*			13,000 mg/kg	-	1,200,000 tons	Poon et al., 1997, Wu et al., 2010, Sarkar et al., 2013, Zhang et al., 2017b, Zhang et al., 2018, Gani and Kazmi 2018, Feng et al., 2020b, Dhar et al., 2023, Zhang et al., 2023b
*Pesticides*							
Imidacloprid (86287518)	*Bacillus*, *Klebsiella*, *Ochrobactrum*, *Pseudomonas*	Used as insecticide in crop protection, horticulture, and fleas control	Cytotoxicity, genotoxicity, neurotoxicity, immunotoxicity and reproductive toxicity	450 mg/kg	0.013 μg/L	600,000 tons	Pandey et al., 2009, Sharma et al., 2014, Pang et al., 2020, Petković Didović et al., 2022, Zhang et al., 2023a
Chlorpyrifos (2730)	*Alcaligenes*, *Bacillus,* *Paracoccus*, *Pseudomonas*	Used as broad-spectrum, insecticide, acaricide and miticide to control foliage- and soil-borne insect pests	Developmental and reproductive toxicity, altered synaptic development, alterations in DNA, RNA, and protein synthesis, inhibition of mitosis	66–223 mg/kg	0.041 μg/L	9,500–10,800 tons	Smegal, 2000, Fu et al., 2024, Nandhini et al., 2021, Ruiz-Arias et al., 2023, Bosu et al., 2024
Carbendazim (25429)	*Bacillus*, *Nocardioides*, *Pseudomonas*, *Ralstonia*, *Rhodococcus*	Used as systemic broad-spectrum fungicide, pre- and postharvest treatment to control the fungal diseases	Induces apoptosis, immunotoxicity and endocrine disturbance in developing embryo, spermatotoxicity, mutagenicity, aneugenicity	>2000 mg/kg	0.1–0.5 μg/L	12,000 tons	Rama et al., 2014, Singh et al., 2016, Bai et al., 2017, Long et al., 2021, Zhou et al., 2023
Alachlor (2078)	*Paracoccus*, *Pseudomonas,* *Rhodococcus*, *Sphingobium*	Used as a selective pre-emergent and post-emergent herbicide to control weeds	Cytotoxicity, mutagenicity, genotoxicity, carcinogenicity, hepatotoxicity, renal toxicity, anemia	930 mg/kg	2 μg/L	3,500–5,000 tons	Zhang et al., 2011, Ghani et al., 2022, Lee and Kim, 2022, Chen et al., 2023
Glyphosate (3496)	*Alcaligenes*, *Bacillus*, *Comamonas*, *Pseudomonas*	Used as non-specific herbicide to control broad range of weeds	Carcinogenicity, induces oxidative stress, genotoxicity, cutaneous toxicity, inhibition of the mitochondrial succinate dehydrogenase activity	2,300 mg/kg	0.1 μg/L	>5,800 tons	Zhan et al., 2018, Singh et al., 2019, Singh et al., 2020, Feng et al., 2020a, Díaz-Soto et al., 2024

‘-’ indicates not reported.

Sources of CECs in the environment can include industrial discharge, improper disposal, excretion of unmetabolized residues, improper sewage management, hospital/laboratory wastewater, agricultural run-off, or processes like eutrophication. Whereas, the prevalence of these compounds depends upon various factors such as industrial activities, agricultural practices, regulatory policies, waste management systems, and environmental conditions (Feng et al., 2023). CEC exposure to humans can occur through various routes like consumer goods, personal care products, ingestion of contaminated food and water, occupational exposure, inhalation of airborne particles and foetal exposure, amongst others, causing a variety of health effects (Feng et al., 2023). For example, long-term exposure to such contaminants has been linked to cancer, endocrine disruption, reproductive tissue damage, immune system suppression, developmental anomalies, and liver damage, amongst other health effects (Radke et al., 2020; Gonsioroski et al., 2020; Lyu et al., 2020; Syafrudin et al., 2021; Balakrishnan et al., 2022; Table 1). Additionally, CECs have been found to bioaccumulate in aquatic biota (Deere et al., 2024) and are toxic to crustaceans (Hossain et al., 2018), earthworms (Gillis et al., 2017), fish (Meador et al., 2016; Yeh et al., 2017; Picó et al., 2019) and molluscs (Canesi et al., 2022), causing ecological disruption. Therefore, removal of these compounds from various ecological compartments is a necessity.

The persistence of CECs in the environment has led to evolution of microbes to utilise them as sole source of carbon and energy (Table 1). Bioremediation involves the application of microbes to clean-up xenobiotics/pollutants from contaminated habitats and provides a desirable alternative to abiotic methods of removal due to its cost-effectiveness, efficiency, and eco-friendliness (Patel et al., 2022). Further, the application of directed genetic engineering approaches, called as “metabolic engineering,” aids in overcoming limitations associated with natural isolates (Dvorak et al., 2017). Additionally, omics techniques have emerged as essential tools for deciphering complex mechanisms underlying CEC biodegradation, which aids in enhancing the understanding of degradation pathways and designing optimal metabolic engineering strategies.

This article aims to provide a comprehensive review of microbial degradation pathways as well as the associated genes and enzymes for four major groups of contaminants of emerging concern (CECs): pharmaceuticals, plasticizers, cyanotoxins, and emerging pesticides (Table 1). Further, the application of omics techniques, including genomics, metagenomics, transcriptomics, proteomics, and metabolomics, to gain system-level insights into the metabolic pathways and regulatory mechanisms driving CEC degradation for development of efficient bioprocess has been described. Additionally, the article also highlights the importance of metabolic engineering strategies to enhance bioremediation efficiency.

## 2 Microbial degradation pathways and genetics

### 2.1 Pharmaceuticals

#### 2.1.1 Antibiotics

Antibiotics are antibacterial agents that function by either killing (bactericidal) or inhibiting the growth (bacteriostatic) of bacteria. Antibiotics are grouped into *beta*-lactams, macrolides, fluoroquinolones, tetracyclines, aminoglycosides, sulfonamides, glycopeptides, oxazolidinones and carbapenems, based on their structure and mechanism of action (Etebu and Arikekpar, 2016). Large scale production, improper sewage management and disposal as well as human excretion of unmetabolized residues contribute to accumulation of these compounds in aquatic and soil ecosystems (Cycoń et al., 2019; Bilal et al., 2020).

Antibiotics have been found to occur in wastewater treatment plants (WWTPs), hospital wastewaters, as well as surface, river and groundwater across the globe. For example, antibiotic concentrations up to 14.5 μg L^−1^ and 64 μg L^−1^ (dominated by *β*-lactams, quinolones and sulfonamides) were detected in hospital effluents and WWTP influents, respectively, in Queensland, Australia. The concentration in surface waters and WWTP effluent was up to 2 and 3.4 μg L^−1^, respectively (Watkinson et al., 2009). *Beta*-lactam antibiotics amoxillin and penicillin G were detected at 13.3–18.47 μg L^−1^ and 3.12–4.75 μg L^−1^ in WWTP influents in Iran (Golchin et al., 2021). Various antibiotic classes such as sulfonamides (285.5–634.9 ng L^−1^), tetracyclines (363.4–753.3 ng L^−1^) and quinolones (1,355.8–1922.4 ng L^−1^) were detected in hospital influents in Xinjiang, China (Li et al., 2016). Sulfonamides (up to 256 ng L^−1^) and quinolones (up to 1,270 ng L^−1^) were detected at high concentrations in Wenyu river in Beijing, China (Liu et al., 2019). Erythromycin has been detected in Korean Municipal WWTP influents at a concentration of 0.4–1 μg L^−1^ (Sim et al., 2010) and 381 ng L^−1^ in River Thurso, Scotland (Nebot et al., 2015). The presence of these compounds in the environment poses a major risk due to the dissemination of antibiotic resistance genes and evolution of resistance phenotype in the microbial community (Li et al., 2015; Rolbiecki et al., 2021; Thakali et al., 2021). Additionally, antibiotic residues cause toxicity to aquatic biota and alter microbial community structure, causing ecological disruption (Ding and He, 2010; Välitalo et al., 2017).

##### 2.1.1.1 Sulfonamides

**FIGURE 1:**
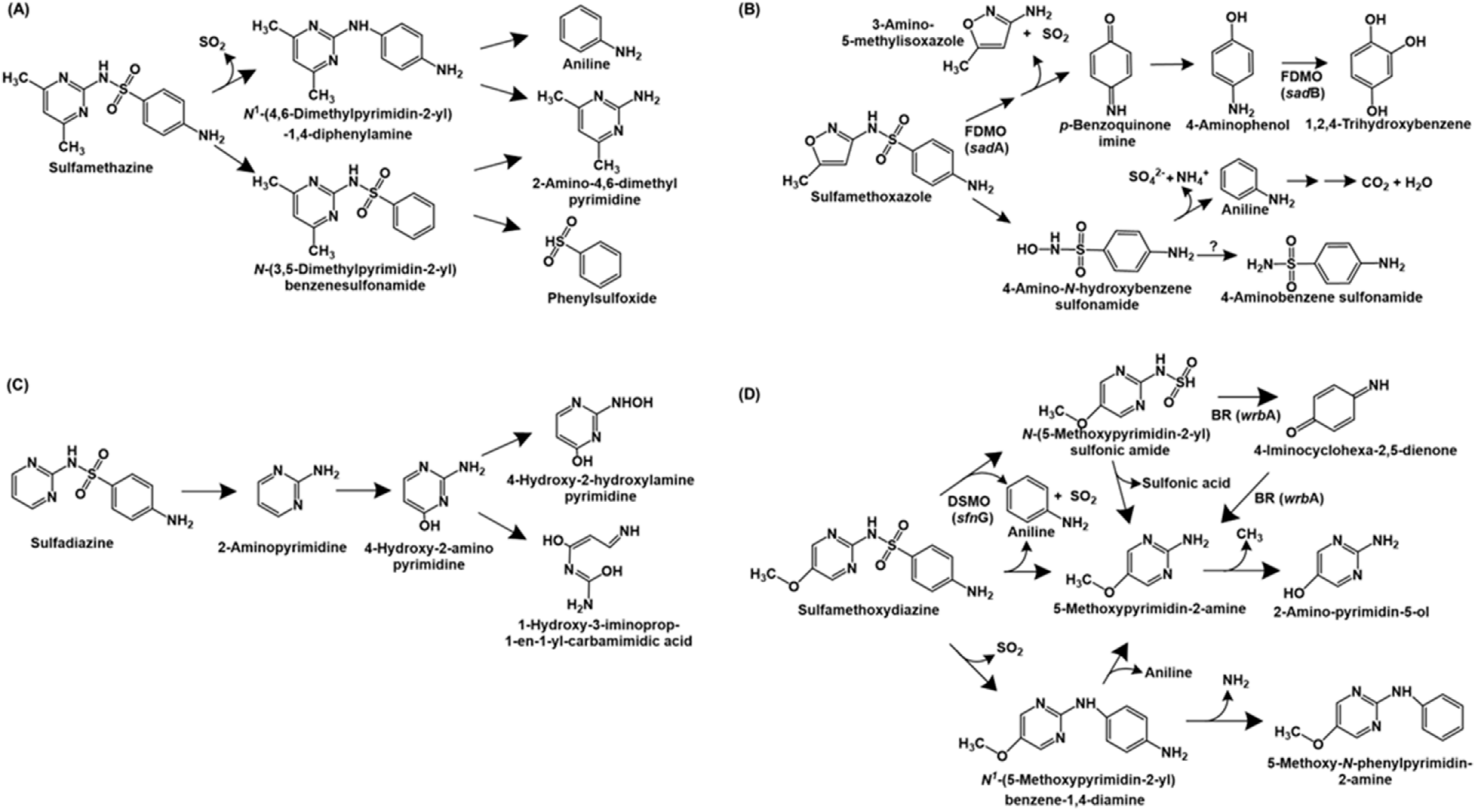
Bacterial degradation pathways of sulfonamide antibiotics: **(A)** sulfamethazine **(B)** sulfamethoxazole **(C)** sulfadiazine and **(D)** sulfamethoxydiazine. Gene encoding of the respective enzymes are indicated in parenthesis. Enzyme abbreviations: FDMO, flavin-dependent monoxygenase; BR, 1,4-benzoquinone reductase; DSMO, dimethylsulfone monoxygenase. Question mark indicates enzyme catalysing reaction not known.

Sulfonamide antibiotics are synthetic antimicrobial agents that are primarily used in human and veterinary medicine to combat bacterial infections. These compounds inhibit the enzyme dihydropteroate synthetase, essential for folic acid synthesis. The bacterium *Bacillus cereus* H38 utilises sulfamethazine as source of carbon, nitrogen and sulphur. The bacterium possesses two pathways for the catabolism of this antibiotic. In pathway I, the S-N bond is cleaved, removing SO_2_ and forming *N*-(4,6-dimethylpyrimidin-2-yl) 1,4-diphenylamine. Further, the C-N bond in this compound is cleaved to form 2-amino-4,6-dimethylpyrimidine and aniline. Pathway II proceeds *via* the cleavage of the *N* ^*4*^ amine bond to form *N*-(3,5-dimethylpyrimidin-2-yl)-benzenesulfonamide, which is also converted to 2-amino-4,6-dimethylpyrimidine and phenyl sulphoxide upon cleavage of the S-N bond (Dong et al., 2022; Figure 1A).

*Pseudomonas psychrophila* HA-4 utilises the antibiotic sulfamethoxazole as the sole source of carbon and energy. The first step of degradation involves the hydrolysis of the compound to 4-amino-*N*-hydroxybenzenesulfonamide and 3-amino-5-methylisoxazole. The former undergoes deamination and desulfurization to form aniline, sulphate and ammonia. Aniline is further metabolised to carbon-di-oxide and water. Alternatively, 4-amino-*N*-hydroxybenzesulfonamide can be converted to 4-aminobenzenesulfonamide (Jiang et al., 2014). The metabolic pathway of sulfomethoxazole in *Microbacterium* sp. BR1 is initiated by *ipso*-hydroxylation to form an unstable intermediate which forms *p*-benzoquinone imine and 3-amino 5-methylisoxazole. The former is reduced to *p*-aminophenol, which undergoes hydroxylation to form 1,2,4-trihydroxybenzene, which might undergo ring-cleavage (Ricken et al., 2015; Ricken et al., 2017; Figure 1B).

The complete metabolic pathways for sulfadiazine and sulfametoxydiazine have been reported in *Arthrobacter* sp. D2 and *Alcaligenes aquatillis* FA, respectively. In strain D2, sulfadiazine is metabolised *via* the cleavage of the sulfonamide bond to form 2-aminopyrimidine, which is hydroxylated at the C-4 position to form 4-hydroxy-2-amino-pyrimidine. Subsequently, this intermediate undergoes ring-opening or hydroxylation of the amine group (to form 4-hydroxy-2-hydroxylamine-pyrimidine; Deng et al., 2016; Figure 1C). Whereas, sulfametoxydiazine metabolism in strain FA has been proposed to proceed *via* three different routes. In pathway I, the breakdown was initiated by the loss of aniline to form *N*-(5-methoxypyrimidin-2-yl) sulfonic amide. This intermediate further forms 5-methoxypyrimidin-2-amine (by loss of sulphonic acid), which forms 2-amino-pyrimidin-5-ol by loss of a methyl group. Alternatively, strain FA can directly form 5-methoxypyrimidin-2-amine by loss of aniline and SO_2_ (pathway II). In pathway III, the loss of SO_2_ from the substrate results in the generation of *N* ^1^-(5-methoxypyrimidin-2-yl) benzene-1,4-diamine. This intermediate can either form 5-methoxypyrimidin-2-amine by cleavage of the C-N bond or 5-methoxy-N-phenylpyrimidin-2-amine by loss of amine group (Du et al., 2023; Figure 1D).

Three sulfonamide degradation genes *sad*A, *sad*B and *sad*C were identified in the genome of *Microbacterium* sp. strain BR1. Both *sad*A and *sad*B encoded flavin-dependent monoxygenases catalysing removal of 3-amino-5-methylisoxazole and SO_2_ from sulfomethoxazole and hydroxylation of 4-aminophenol to trihydroxybenzene, respectively. Whereas *sad*C encoded a FMN reductase involved in delivering reduced FMN to SadA and SadB. Similar homologues of *sad*ABC have been found in the genomes of other *Actinobacteria* such as *Paenarthrobacter* sp. A01 (Cao et al., 2019), *Leucobacter sulfamidivorax* (Reis et al., 2019) and *Arthrobacter* sp. D2 and D4 (Deng et al., 2016). Aside from *Actinobacteria*, *Alcaligenes aquatillis* FA harbored three sulfametoxydiazine metabolic genes: *wrb*A encoding 1,4-benzoquinone reductase (involved in formation or degradation of 4-iminocyclohexa-2,5-dienone), *pca*C encoding 4-carboxymuconolactone decarboxylase (involved in aromatic ring processing) and *sfn*G encoding dimethylsulfone monooxygenase (involved in hydroxylation of sulfametoxydiazine). Further, *dfr*A26 (dihydrofolate reductase) and *sul2* (dihydropteroate synthetase) genes were hypothesised to be involved in resistance to sulfonamides in strain FA (Du et al., 2023).

##### 2.1.1.2 Beta-lactams

**FIGURE 2:**
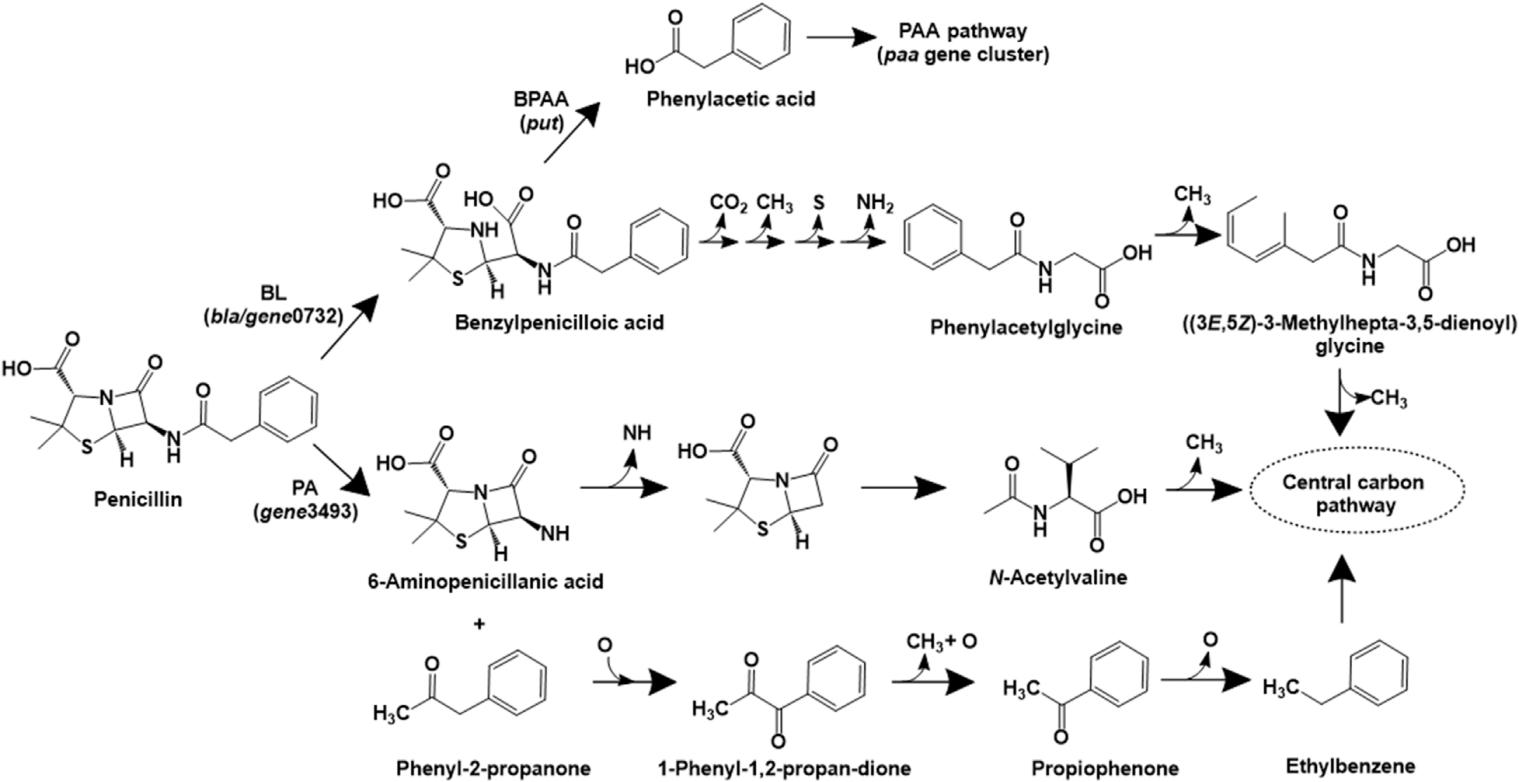
Bacterial degradation pathways of penicillin. Gene encoding of the respective enzymes are indicated in parenthesis. Multiple arrows indicate multiple metabolic steps. Enzyme abbreviations: BL, *beta*-lactamase; PA, penicillin acylase; BPAA, benzylpenicilloic acid amidase.

*Beta*-lactam antibiotics consist of a characteristic beta-lactam ring and function by inhibiting bacterial cell wall synthesis. Although biotransformation products have been reported for various beta-lactams such as imipenem (Minerdi et al., 2016), and ampicillin (Zumstein and Helbling, 2019), the complete mineralisation pathway has been only reported for penicillin G. The penicillin G mineralisation pathway has been detailed in proteobacterial isolates belonging to the genera *Burkholderia* spp. (strain ABC02), *Pseudomonas* spp. (ABC07), *Pandoraea* spp. (strains ABC08 and ABC10). Initially, the enzyme beta-lactamase converts penicillin to benzylpenicilloic acid, which was acted upon by an amidase or a hydrolase type of enzyme to form phenylacetic acid. Subsequently, this intermediate is converted into acetyl-CoA and succinyl-CoA (central carbon intermediates) *via* the phenylacetate pathway, conserved amongst various isolates (Figure 2). The genomic analyses revealed that strain ABC07 carries two major operons for penicillin catabolism, the *put* and *paa* operon. While the *put* operon encodes *beta*-lactamase (*bla*), major superfamily transporter (*mfs*) and amidases (*put*1 and *put*2), the *paa* operon encodes enzymes involved in phenylacetic acid catabolism. Similar genes were also detected in strains ABC02, ABC08 and ABC10 (Crofts et al., 2018).

In *Sphingobacterium* sp. SQW1, three different pathways for degradation of penicillin G sodium have been proposed (Zhang et al., 2024). In the intracellular pathway, penicillin is converted to benzylpenicilloic acid, which undergoes decarboxylation, demethylation, desulfurization and deamination reactions to form phenylacetylglycine. This intermediate undergoes ring opening and multiple demethylation reactions to form central carbon intermediates (Figure 2). A similar pathway involving the action of extracellular beta-lactamase (to form benzylpenicilloic acid) and multiple demethylation, desulfurization and deamination reactions has also been proposed (Zhang et al., 2024). An alternative extracellular pathway involves the action of the enzyme penicillin acylase (on penicillin) to form phenyl-2-propanone and 6-aminopenicillanic acid (6-APA) by an acylation decarboxylation reaction. The former compound undergoes oxidative dehydrogenation to form 1-phenyl-1,2-propanedione, which ultimately forms carbon-di-oxide and water. Whereas, 6-APA undergoes deamidation, hydrolysis of the beta-lactam ring, ring–opening desulfurization and demethylation to form *N*-acetylvaline, which undergoes demethylation and ultimately forms carbon-di-oxide and water (Figure 2). The genes encoding beta-lactamase (*gene*0732) and penicillin amidase (*gene*3493) were detected in strain SQW1 (Zhang et al., 2024).

##### 2.1.1.3 Erythromycin

**FIGURE 3:**
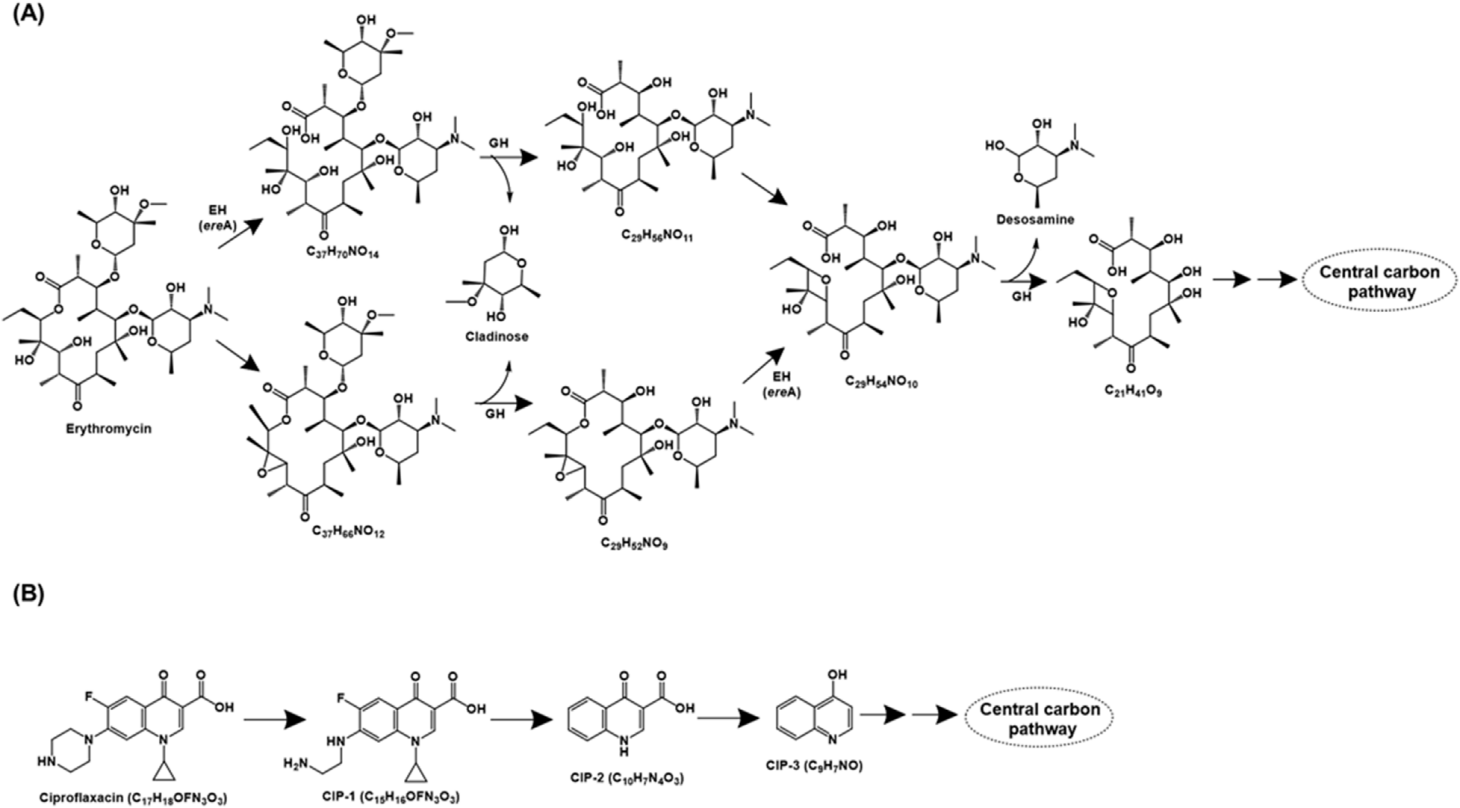
Bacterial degradation pathways of **(A)** erythromycin and **(B)** ciproflaxacin. Genes encoding of the respective enzymes are indicated in parenthesis. Multiple arrows indicate multiple metabolic steps. Enzyme abbreviations: EH, erythromycin hydrolase; GH, glycoside hydrolase.

Erythromycin, a macrolide class of antibiotic, consists of a characteristic macrocyclic lactone ring and functions by inhibiting bacterial protein synthesis *via* binding to 50S ribosomal subunit. Two major erythromycin mineralisation pathways have been detailed in *Paracoccus versutus* W7. In the first pathway, the antibiotic is acted upon by the esterase EreA (Erythromycin hydrolase), leading to opening of the lactone ring. The intermediate generated (C_37_H_70_NO_14_) was cleaved by glucoside hydrolase, resulting in removal of the cladinose moiety. Further, dehydration followed by the action of glycoside hydrolase results in the removal of desosamine moiety. The remaining main chain compound (C_21_H_41_O_9_), cladinose and desosamine are metabolised *via* tricarboxylic acid cycle (TCA). Alternatively, erythromycin was converted to the intermediate C_37_H_66_NO_12_ and further acted upon by glucoside hydrolase to catalyse the removal of cladinose. The generated intermediate (C_29_H_52_NO_9_) is acted upon by EreA to form C_29_H_54_NO_10_. The action of glucoside hydrolase generates C_21_H_41_O_9_ and desosamine, which are funnelled into the central carbon metabolism (Ren et al., 2023a; Figure 3A). A similar erythromycin metabolism pathway has been proposed in *Rhodococcus gordoniae* rjjtx-2 (Ren et al., 2022).

Literature on the genetic background of erythromycin catabolism has mainly focussed on the hydrolase *ere* genes, while reports about other enzymes/genes are scanty. The first erythromycin esterase gene *ere*A was identified in *Escherichia coli* (Ounissi and Courvalin, 1985) and its homologs have been detected in genus like *Rhodococcus* (Ren et al., 2022), *Paracoccus* (Ren et al., 2023a) and *Providencia* (Plante et al., 2003), amongst others. Similarly, type-II erythromycin hydrolase, encoded by *ere*B was detected in *E. coli* (Arthur et al., 1986) and its homologs have been frequently detected in environmental isolates such as *Staphylococcus* (Schmitz et al., 2000), *Klebsiella* and *Salmonella* (Fuentes et al., 2014), amongst others. The newly discovered *ere*C and *ere*D are less prevalent and have been detected in *Klebsiella* (Yong et al., 2009) and *Riemerella* (Xing et al., 2015), respectively. Interestingly, the *ere* homologs (except for *ere*D) are associated/localised on mobile genetic elements that aid in their distribution in the microbial community *via* horizontal gene transfer (Arthur et al., 1986; Biskri and Mazel, 2003; Yong et al., 2009).

##### 2.1.1.4 Ciproflaxacin

Ciproflaxacin is a fluoroquinolone type of antibiotic that functions by inhibiting the bacterial enzyme DNA gyrase (topoisomerase II) and topoisomerase IV. The ciprofloxacin metabolic pathway has been proposed for consortium XG consisting of bacteria belonging to genera *Achromobacter*, *Bacillus*, *Lactococcus*, *Ochrobactrum* and *Enterococcus*. Ciproflaxacin (C_17_H_18_OFN_3_O_3_) degradation is initiated by the loss of the C_2_H_2_ moiety from the piperazine ring to form CIP-1 (C_15_H_16_OFN_3_O_3_). Further, the loss of piperazine moiety, cyclopropyl, and fluorine atom results in the formation of CIP-2 (C_10_H_7_N_4_O_3_), which subsequently forms CIP-3 (C_9_H_7_NO) by decarboxylation. CIP-3 was further mineralised to CO_2_, H_2_O, NH_4_^+^, NO_3_^−^ and F^−^ by the consortia XG (Figure 3B; Feng et al., 2019).

##### 2.1.1.5 Chloramphenicol

**FIGURE 4:**
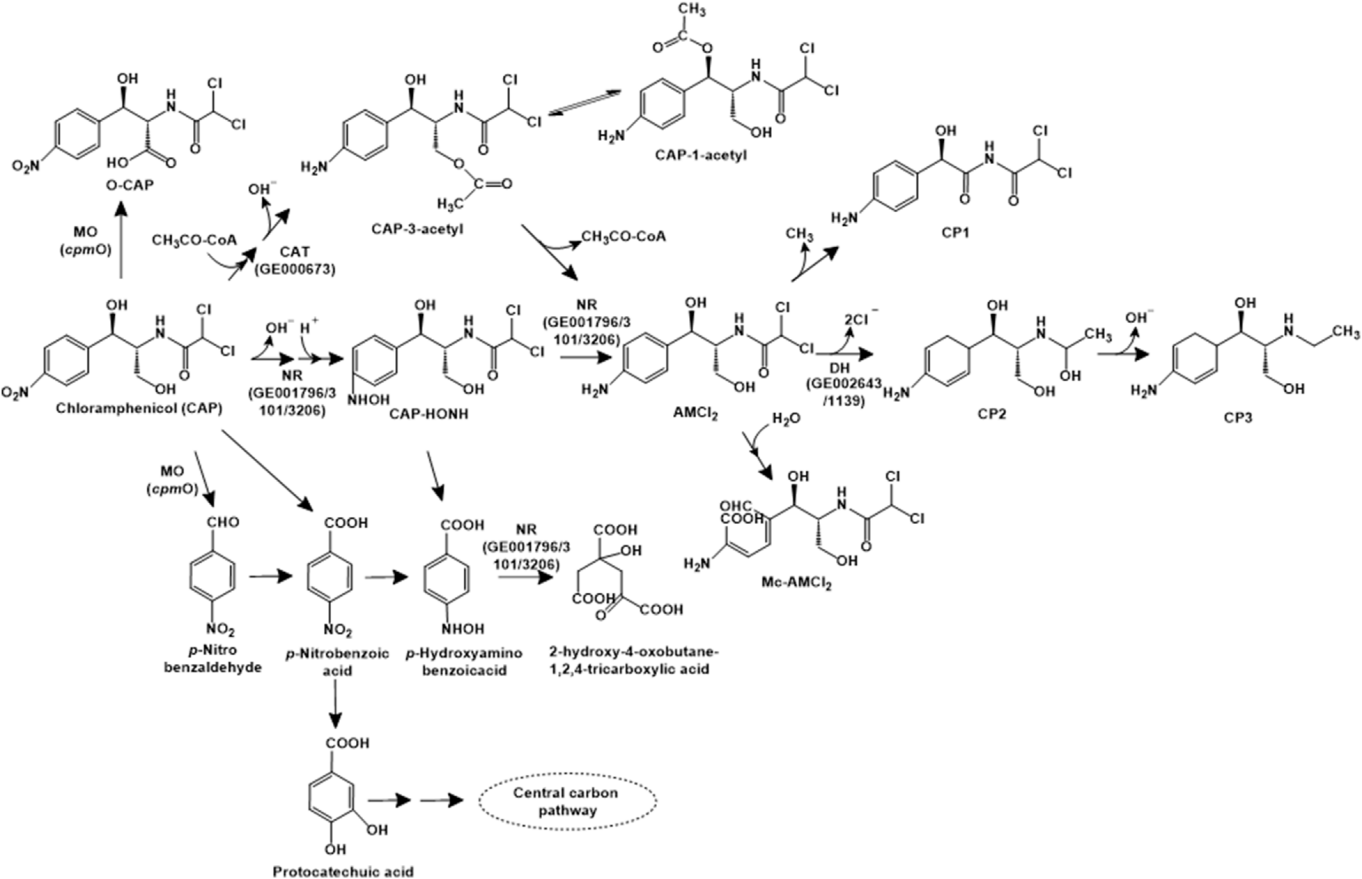
Bacterial degradation pathways of chloramphenicol. Enzyme abbreviations: MO, multifunctional oxidase; CAT, chloramphenicol O-acetyltransferase type B; NR, nitroreductase; DH, haloacid or haloalkane dehalogenase. Gene encoding of the respective enzymes are indicated in parenthesis. Multiple arrows indicate multiple metabolic steps.

Chloramphenicol is a broad-spectrum antibiotic that binds 50S ribosomal subunit and inhibits protein synthesis. In *Aeromonas media* SZW-3, chloramphenicol has been reported to be catabolised by three major pathways. The first pathway involves the cleavage of the bond between the side chain of C1 and C2, leading to the formation of *p-*nitrobenzoic acid, which is oxidised to form *p-*hydroxyaminobenzoic acid, which is further ring-cleaved (Figure 4). In pathway II, the nitro-group is sequentially reduced to an amino group, forming AMCl_2_. This intermediate can further be demethylated (to form CP1), dechlorinated (to form CP2) or ring-cleaved (to form Mc-AMCl_2_). Alternatively, chloramphenicol can undergo acetylation and sequential reduction of the nitro group to an amine, following a route similar to pathway I (Figure 4). The genomic analyses of strain SDW-3 identified genes *rar*D (encoding a permease that provides resistance), chloramphenicol O-acetyltransferase type B encoding gene (GE000673; involved in the acetylation of chloramphenicol), three nitro-reductases (GE003101, GE001796, GE003206; involved in biodetoxification) as well as haloacid and haloalkane dehalogenases (GE002643 and GE001139; involved in dechlorination) (Tan Z. et al., 2022).

*Sphingobium* sp. WTD-1 has been reported to utilise this antibiotic as the sole source of carbon and energy. Three metabolic pathways for chloramphenicol have been proposed in strain WTD-1. The first pathway involves the acetylation to chloramphenicol-3-acetyl (CAP-3-acetyl), which is non-enzymatically converted to chloramphenicol-1-acetyl (CAP-1-acetyl). The second pathway involves the dehydrogenation at the C-3 hydroxyl group to form 2,2-dichloro-*N*-(1,1,3-trihydroxy-3-(4-nitrophenyl) propan-2-yl) acetamide (DHNOA), which is further oxidised to the dead-end metabolite 2-(2,2-dichloroacetamido)3-hydroxy-3-(4-nitrophenyl) propanoic acid (O-CAP). The third pathway involves cleavage of the C1-C2 bond to form *para*-nitrobenzaldehyde (PNBD), which is converted to *para-*nitrobenzoic acid (PNBA) and further, protocatechuic acid. This intermediate undergoes *ortho-* or *meta*-ring cleavage to form TCA cycle intermediates (Gao et al., 2024a; Figure 4). A novel multifunctional oxidase, CpmO, which carries out the oxidation of C-3 hydroxyl as well as cleavage of C1-C2 bond was identified in the genome of strain WTD-1, purified and characterised (Gao et al., 2024b).

#### 2.1.2 Analgesics

Analgesics are pain-relieving medications that can be categorized into two groups: opioid and non-opioid. Opioid analgesics function by impacting pain perception in the brain by affecting ion channels or receptors, while non-opioid analgesics inhibit prostaglandin synthesis. Amongst these, non-opioid analgesics such as ibuprofen, acetaminophen and naproxen are widely prescribed and prevalent, and are therefore the subject of current discussion. The easy (over the counter) availability, low toxicity, extensive use, improper disposal and excretion of unmetabolized drug/associated metabolites have contributed to accumulation of these analgesics in the environment, causing adverse effects to biota, especially aquatic ecosystems (Parolini, 2020; Jan-Roblero and Cruz-Maya, 2023). For example, ibuprofen has been detected in influents (5–22 μg L^−1^) and effluents (0.1–2 μg L^−1^) of WWTPs in south-western India (Praveenkumarreddy et al., 2021). Acetaminophen (4.4–9.2 μg L^−1^), ibuprofen (0.8–1.2 μg L^−1^) and naproxen (0.5–0.9 μg L^−1^) have been detected at varying concentrations in municipal WWTPs in Korea (Sim et al., 2010). Acetaminophen (22.8 μg L^−1^) has been detected at high concentrations in the water of River Thurso, Scotland (Nebot et al., 2015). The concentration of naproxen ranged from 20–231 ng L^−1^ and 13–80 ng L^−1^ in influents and effluents, respectively of Italian WWTPs (Patrolecco et al., 2015). The occurrence of these compounds at reported concentrations causes significant toxicity to aquatic biota (Ragugnetti et al., 2011; Aguirre-Martínez et al., 2015), thereby leading to ecological disruption.

##### 2.1.2.1 Ibuprofen

**FIGURE 5:**
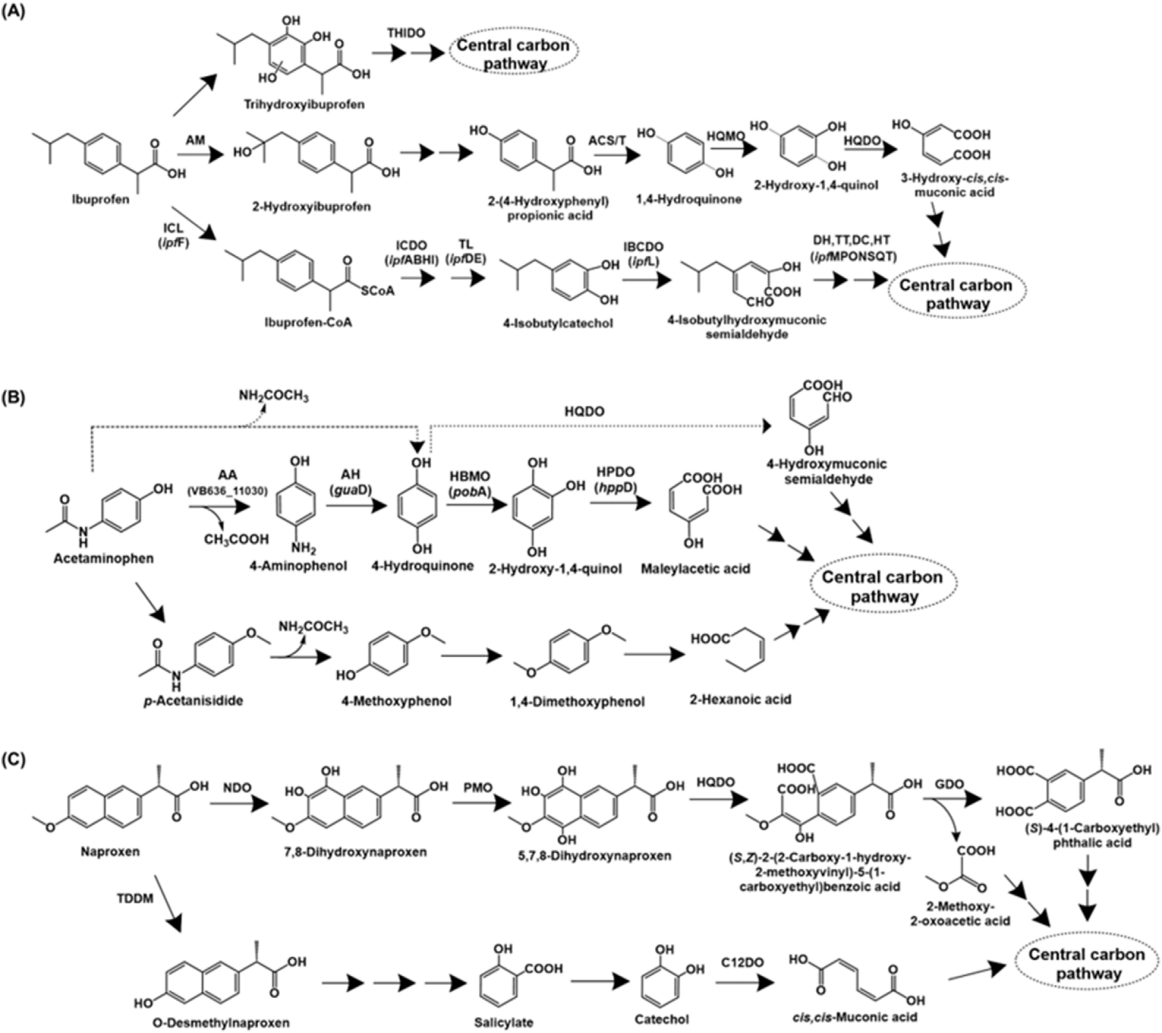
Bacterial degradation pathways of analgesics **(A)** ibuprofen **(B)** acetaminophen and **(C)** naproxen. Genes encoding respective enzymes are indicated in parenthesis. Multiple arrows indicate multiple metabolic steps. Enzyme abbreviations: THIDO, trihydroxyibuprofen dioxygenase; AM, aliphatic monoxygenase; ACS/T, acyl-CoA synthase/thiolase; HQMO, 1,4-hydroquinone monoxygenase; HQDO, hydroxyquinol-1,2-dioxygenase; ICL, ibuprofen CoA ligase; ICDO, ibuprofen-CoA dioxygenase; TL, thiolase; IBCDO, isobutylcatechol dioxygenase; DH, dehydrogenase; TT, tautomerase; DC, decarboxylase; HT, hydratase; AA, M20 aminoacylase family aminohydrolase; AH, aminohydrolase or guanidine deaminase; HBMO, 4-hydroxybenzoate 3-monoxygenase; HPDO, hydroxyquinol dioxygenase or 4-hydroxyphenylpyruvate dioxygenase; TDDM, tetrahydrofolate-dependent O-demethylase; NDO, naphthalene dioxygenase; PMO, phenol monoxygenase; GDO, gentisate dioxygenase; C12DO, catechol-1,2-dioxygenase.

Ibuprofen [2-(*p*-isobutylphenyl) propionic acid] is a widely used non-steroidal anti-inflammatory drug (NSAID) that functions by inhibiting the enzyme cyclooxygenase (COX), involved in prostaglandin biosynthesis (Ghlichloo and Gerriets, 2023). The complete degradation pathway of ibuprofen has been described for *Bacillus thuringiensis* B1. The first step involves the aliphatic side-chain hydroxylation to form 2-hydroxyibuprofen by the action of an aliphatic monooxygenase. This intermediate is converted to 2-(4-hydroxyphenyl-) propionic acid, which is acted upon by acyl-CoA synthase/thiolase to form 1,4-hydroquinone. The action of 1,4-hydroquinone monooxygenase forms 2-hydroxy-1,4-quinol, which undergoes *ortho*-ring cleavage by the action of hydroxyquinol 1,2-dioxygenase to form 3-hydroxy-*cis,cis*-muconic acid, which is funnelled into central carbon pathway (Marchlewicz et al., 2017; Figure 5A).

Alternatively, ibuprofen is also catabolised *via* aromatic ring hydroxylation and cleavage. *Sphingomonas* sp. Ibu-2 metabolises ibuprofen to ibuprofen-CoA by the action of a CoA-ligase. Further, this intermediate is converted to isobutylcatechol (upon removal of propionic acid side-chain), which is ring-cleaved *via meta* route (Murdoch and Hay, 2013). In *Variovorax* sp. strain Ibu-1, ibuprofen is metabolised *via* the formation of trihydroxyibuprofen, which has been proposed to undergo *meta*-ring cleavage to form aliphatic intermediates (Murdoch and Hay, 2015; Figure 5A).

The genomic sequence of strain Ibu-2 revealed the presence of *ipf*ABDEF gene cluster involved in degradation, along with genes *ipf*HI. The genes encoded ibuprofen CoA-ligase (IpfF), ibuprofen-CoA dioxygenase (IpfABHI), thiolase (IpfD) involved in removal of acyl-CoA group and IpfE (unkown function; involved in the generation of isobutylcatechol) (Żur et al., 2018). Similar gene clusters for the conversion of ibuprofen to isobutylcatechol have been identified in *Sphingopyxis granuli* RW412 (Aguilar-Romero et al., 2021) and *Rhizorhabdus wittichii* MPO218 (Aulestia et al., 2022). Additionally, the genes for further metabolism of isobutylcatechol, that is, *ipf*L (4-isobutylcatechol-2,3-dioxygenase) and *ipf*M (hydroxymuconic semialdehyde dehydrogenase), *ipf*P (tautomerase), *ipf*O (decarboxylase), *ipf*N (hydratase), *ipf*S (hydratase), *ipf*Q (aldehyde dehydrogenase) and *ipf*T (acyl-CoA dehydrogenase) have been identified in strain MPO218. Further, the upper pathway genes were flanked by IS6100 insertion elements, indicating probable acquisition by horizontal gene transfer (Aulestia et al., 2022).

##### 2.1.2.2 Acetaminophen

Paracetamol, also known as acetaminophen [*N*-(4-hydroxyphenyl)acetamide], is a commonly used analgesic and antipyretic that functions by inhibiting prostaglandin synthesis (Roberts et al., 2016). The first step of bacterial acetaminophen degradation proceeds *via* the action of aryl acyla midase to form 4-aminophenol, which is further converted to hydroquinone by the action of an aminohydrolase. Hydroquinone formation has also been proposed to occur directly with release of acetamide as a byproduct (Hu et al., 2013). Hydroquinone undergoes ring-cleavage by the action of a dioxygenase to form organic acids (Hu et al., 2013; Żur et al., 2018). Alternatively, hydroquinone can undergo hydroxylation to form 1,2,4-trihydroxybenzene, followed by ring-cleavage (Takenaka et al., 2003; Figure 5B). An alternate pathway for paracetamol degradation has been proposed in soil micro-organisms by Li et al., involving the methylation of paracetamol to *para*-acetanisidide, which is converted to 4-methoxyphenol and further, 1,4-dimethoxybenzene. This intermediate is further ring-cleaved to aliphatic intermediates (Li et al., 2014; Figure 5B).

The genetics of paracetamol degradation were detailed in the bacterium *Paracoccus* sp. APAP_BH8. The genes encoding a M20 aminoacylase family aminohydrolase (involved in hydrolysis of paracetamol to 4-aminophenol), *gua*D (guanidine deaminase for the formation of hydroquinone from 4-aminophenol), *pob*A (4-hydroxybenzoate-3-monooxygenase for conversion of hydroquinone to hydroquinol) and *hpp*D (4-hydroxyphenylpyruvate dioxygenase for the ring-cleavage of hydroquinol) were identified (Pandey et al., 2024). Amidase genes involved in the conversion of paracetamol to 4-aminophenol were detected in two *Pseudomonas* species, with mobile genetic elements in their vicinity, indicating probable role of horizontal gene transfer. The extradiol dioxygenase genes involved in subsequent degradation were also detected in the genome (Rios-Miguel et al., 2022).

##### 2.1.2.3 Naproxen

Naproxen [6-methoxy-alpha-methyl-2-naphthaleneacetic acid] is an NSAID, widely used as an analgesic and antipyretic. Similar to ibuprofen, naproxen functions by suppressing the cyclooxygenase (COX) enzyme activity (Ríos et al., 2022). The complete naproxen degradation pathways have been described in *Stenotrophomonas maltophilia* KB2 and *Bacillus thuringiensis* B1. In strain KB2, naproxen is dihydroxylated to 7,8-dihydroxynaproxen by the action of naphthalene dioxygenase, which has been reported to have a wide substrate range (Lee and Gibson, 1996; Selifonov et al., 1996; Phale et al., 2007). Further, the action of phenol monooxygenase generates 5,7,8-trihydroxynaproxen, which undergoes ring-fission by the action of hydroxyquinol 1,2-dioxygenase, leading to the generation of an monoaromatic intermediate. Further, the action of gentisate dioxygenase results in conversion to an aliphatic intermediate, which is funnelled into the central carbon metabolism (Wojcieszyńska et al., 2014; Figure 5C).

Naproxen degradation in *Bacillus thuringiensis* B1 involves the removal of methyl group to form *O*-desmethylnaproxen by the action of tetrahydrofolate-dependent *O*-demethylase. This intermediate is converted to salicylate, which subsequently forms either catechol or gentisate (dihydroxy-intermediates). The major naproxen degradation proceeds *via* ring-cleavage of catechol by enzyme catechol-1,2-dioxygenase; whereas, ring-cleavage by the enzymes gentisate-1,2-dioxygenase (acting on gentisate) and salicylate-1,2-dioxygenase (acting on salicylate) are minor pathways (Górny et al., 2019; Figure 5C).

#### 2.1.3 Steroid sex hormones

In humans, steroid sex hormones are synthesised from cholesterol and can be classified as androgens, progestogens and oestrogens based on their structure and function. Androgens (such as testosterone) and oestrogens (such as oestrone: E1, 17*β*-oestradiol: E2, estriol: E3) regulate the development and maintenance of secondary sexual characteristics as well as the reproductive system in males and females, respectively. Whereas, progestogens (like progesterone) are essential for implantation of the embryo and maintenance of pregnancy. Aside from endogenous (naturally occurring) sex hormones, synthetic derivatives of androgens (like 19-nortestosterone), oestrogens (like 17α-ethynyloestradiol: EE2) and progestogens (like progestin) find application in agriculture, aquaculture as well as human health (Chiang et al., 2020).

Major sources of these hormones in the biosphere include human and animal excreta (Lange et al., 2002; Chang et al., 2011), use of manure and sewage derivatives as fertilizers (Kjaer et al., 2007; Hamid and Eskicioglu, 2012) and microbial transformation of phytosterols (Orrego et al., 2009). These compounds have been detected at varying concentrations in the environment. For example, the influent concentration of natural androgens was found to be 2,977 ± 739 ng L^−1^ (androsterone), 640 ± 263 ng L^−1^ (epiandrosterone) and 270 ± 132 ng L^−1^ (androstenedione) in WWTPs in Beijing, China (Chang et al., 2011). The concentration of Estrone (E1) was found to be 5.4–25 ng L^−1^ in Swiss hospital wastewater (Zhang et al., 2017a). The oestrogens Estrone (E1), 17*β*-estradiol (E2), and 17α-ethynylestradiol (EE2) were detected in raw sewage at concentrations up to 104, 66.9, and 5.7 ng L^−1^, respectively in Ontario, Canada (Atkinson et al., 2012). The persistence of these compounds in the environment impacts fish, amphibians and mammals as these hormones function as endocrine disruptors (Aris et al., 2014) and pheromone mimics (Doyle and Meeks, 2018). As compared to androgens and oestrogens, the bacterial degradation of progestogens is poorly detailed, with only biotransformation products being reported (Chiang et al., 2020).

##### 2.1.3.1 Testosterone

**FIGURE 6:**
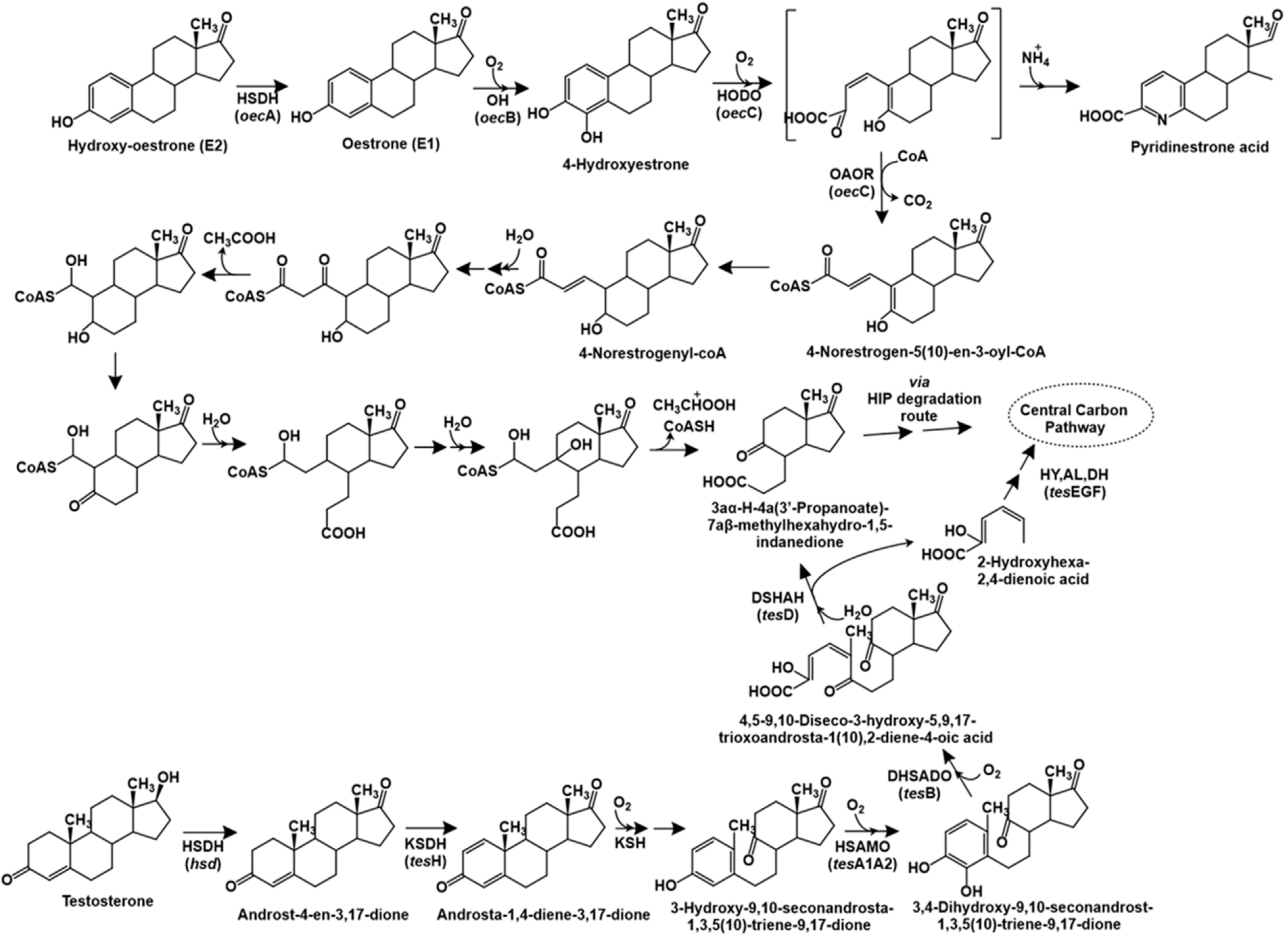
Bacterial degradation pathways of testosterone and oestrone. Genes encoding respective enzymes are indicated in parenthesis. Multiple arrows indicate multiple metabolic steps. Enzyme abbreviations: HSDH, 17*β*-oestradiol dehydrogenase; OH, oestrone 4-hydroxylase; HODO, 4-hydroxyestrone 4,5-dioxygenase; OAOR, 2-oxoacid oxidoreductase; HSDH, hydroxysteroid dehydrogenase; KSDH, ketosteroid dehydrogenase; KSH, 3-ketosteroid 9α-hydroxylase; HSAMO, 3-hydroxy-9,10-secoandrosta-1,3,5 (10)-triene-9,17-dione hydroxylase; DHSADO, 3,4-dihydroxy-9,10-secoandrosta-1,3,5 (10)-triene-9,17-dione dioxygenase; DSHAH, 4,5–9,10-diseco-3-hydroxy-5,9,17-trioxoandrosta-1 (10),2-dien-4-oic acid hydrolase; HY, (2Z,4Z)−2-hydroxyhexa-2,4-dienoic acid hydratase; AL, aldolase; DH, acetoaldehyde dehydrogenase.

The degradation of testosterone has been primarily studied in *Comamonas testosteroni* through the 9,10-seco pathway. The first step of this catabolic pathway involves the oxidation of the 17-hydroxyl group to a carbonyl group to form androst-4-en-3,17-dione (AD), catalysed by the enzyme 17*β*-hydroxysteroid dehydrogenase. Further, the action of 3-ketosteroid dehydrogenase (TesH) introduces a double bond between C-1 and C-2 to form androsta-1,4-diene-3,17-dione (ADD), which is further hydroxylated at C-9 position by the enzyme 3-ketosteroid 9α-hydroxylase to form 9α-hydroxy-androsta-1,4-diene-3,17-dione. This intermediate is unstable and forms 3-hydroxy-9,10-seconandrosta-1,3,5 (10)-triene-9,17-dione (3-HSA) upon spontaneous cleavage of the bond between C-9 and C-10 and aromatisation (Figure 6). Further, the aromatic ring is hydroxylated by the TesA1A2 monooxygenase to form the catecholic intermediate 3,4-dihydroxy-9,10-seco nandrost-1,3,5 (10)-triene-9,17-dione (3,4-DHSA), which undergoes *meta* ring-cleavage by the action of TesB extradiol dioxygenase to form 4,5–9,10-diseco-3-hydroxy-5,9,17-trioxoandrosta-1 (10),2-diene-4-oic acid (4,9-DSHA). This intermediate undergoes hydrolytic cleavage between C-5 and C-10 to produce 3aα-H-4a (3′-propanoate)-7a*β*-methylhexahydro-1,5-indanedione (HIP) and 2-hydroxyhexa-2,4-dienoic acid. The latter is further metabolised by the action of hydratase (TesE), aldolase (TesG) and a dehydrogenase (TesF). The HIP intermediate is reported for various steroid hormone degradation pathways and multiple bacteria possess a common HIP degradation pathway (Chiang et al., 2020; Figure 6). The genes involved in testosterone metabolism in *C. testosteroni* TA441 have been reported to be localised as a 120 kb mega cluster carrying the aromatic ring-degradation genes (*tes*GFEDA1A2HIJ-*scd*A) involved in catabolism of A and B rings and the *β*-oxidation gene cluster (*ste*ABCD-*tes*B-*scd*L1L2NKYM1M2FE-25–26-EC1C2GDJ-*tes*R) involved in HIP degradation (C and D rings). The genes encoding 3α-hydroxydehydrogenase (3α-DH) and 3-ketosteroid Δ4-5 isomerase (*ksi*) are localised between the two clusters. The *tes*R gene encoded a positive regulator of both the gene clusters (Horinouchi and Hayashi, 2023).

##### 2.1.3.2 Oestrogens

The complete degradation pathway of oestrogen (E1: oestrone) has been proposed for *Sphingomonas* sp. strain KC8 *via* the 4,5-seco route (Wu et al., 2019). The first step involves the hydroxylation of oestrogen to 4-hydroxyestrone. This catecholic intermediate undergoes *meta* ring-cleavage by the action of 4-hydroxyestrone 4,5-dioxygenase. The product of this reaction is unstable; and undergoes abiotic recyclization (in presence of ammonium) to form pyridinestrone acid as a dead-end product. Alternatively, the enzyme 2-oxoacid oxidoreductase (belonging to indolepyruvate ferredoxin oxidoreductase family) catalyses the removal of C-4 (as CO_2_) and adds a coenzyme-A (CoA) moiety to the C-3 carbon to form the intermediate 4-norestrogen-5 (10)-en-3-oyl-CoA through oxidative decarboxylation, which undergoes reduction to 4-norestrogenyl-CoA. The C-2 and C-3 carbons (part of the A-ring) are removed *via* thiolytic *β*-oxidation by the action of enzymes enoyl-CoA hydaratase, *β*-hydroxyacyl-CoA dehydrogenase and thiolase. Further, the B-ring of oestrone undergoes hydrolytic cleavage, followed by aldolytic cleavage to remove C-1 and C-10, resulting in the formation of HIP, which is metabolised *via* the HIP degradation pathway (Wu et al., 2019; Chiang et al., 2020; Figure 6).

The genome of strain KC8 has been reported to harbour the gene *oec*A (3*β*,17*β*-hydroxysteroid dehydrogenase) and three other clusters for the metabolism of oestrogen. The cluster I carries the gene *oec*B encoding flavin-dependent estrone-4-hydroxylase which converts estrone to 4-hydroxyestrone. Whereas, cluster II carries the *oec*C gene encoding 4-hydroxyestrone-4,5-dioxygenase and other genes involved in *β*-oxidation. Whereas, cluster III encodes enzymes involved in C/D ring degradation (Chen et al., 2017).

#### 2.1.4 Antidepressants

##### 2.1.4.1 Fluoxetine

Fluoxetine [*N*-methyl-3-phenyl-3-[4-(trifluoromethyl)phenoxy]propan-1-amine] (sold under the brand name Prozac) is an antidepressant belonging to the class of selective serotonin reuptake inhibitors (SSRI). Due to its widespread application in treatment of psychiatric disorders, it has been frequently detected in aquatic ecosystems, causing toxicity to biota (Brooks et al., 2003; Shi et al., 2019; Deere et al., 2021; Ma et al., 2022). For example, fluoxetine has been detected in WWTPs and receiving waters of the Huangpu River, China at concentrations upto 42.9 ng L^−1^ (Wu et al., 2017). Fluoxetine and its human metabolite, norfluoxetine were detected at a concentration of 3.5–16 ng L^−1^ in raw wastewater and 1.2–15 ng L^−1^ in treated wastewater in Uppsala, Sweden (Barclay et al., 2012).

**FIGURE 7:**
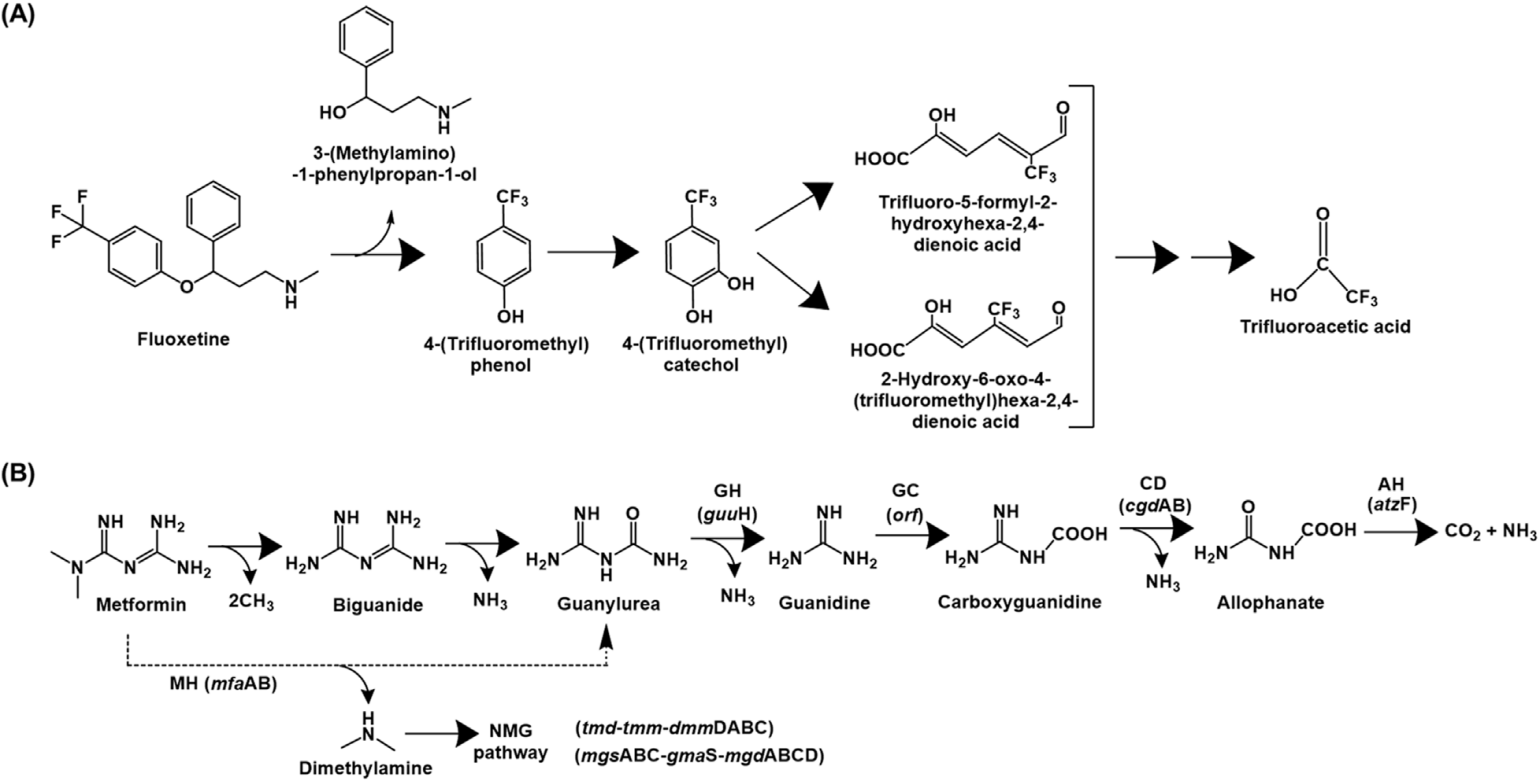
Bacterial degradation pathways of **(A)** fluoxetine and **(B)** metformin. Genes encoding respective enzymes are indicated in parenthesis. Multiple arrows indicate multiple metabolic steps. Enzyme abbreviations: MH, metformin hydrolase; GH, guanylurea hydrolase; GC, guanidine carboxylase; CD, carboxyguanidine deaminase; AH, allophanate hydrolase; NMG pathway, *N*-methylglutamate pathway.

Fluoxetine catabolic pathway has been detailed for various *Bacillus* spp., *Pseudomonas* spp. and *Comamonas testosteroni*, which utilised it as the sole source of carbon and energy (Khan and Murphy, 2021). The fluoxetine degradation is initiated by hydrolysis of the ether bond to yield 4-(trifluoromethyl) phenol (TFMP) and 3-(methylamino)-1-phenylpropan-1-ol. The latter is utilised as the sole source of carbon and energy while TFMP was accumulated in the culture medium. However, the strains exhibited growth on TFMP as sole carbon source, which was further hydroxylated to 4-(trifluoromethyl)catechol. This intermediate was ring-cleaved *via* the *meta* pathway, as indicated by the presence of specific metabolites in the culture medium. These aliphatic intermediates undergo subsequent decarboxylation, aldolytic cleavage, hydroxylation, oxidation and a final decarboxylation to form trifluoroacetic acid, which was a dead-end product. Additionally, fluoride ion was also detected in the culture medium due to defluorination *via* photolytic degradation of the *meta* ring-cleavage product (Figure 7A; Khan and Murphy, 2021).

#### 2.1.5 Antidiabetics

##### 2.1.5.1 Metformin

Metformin (*N*,*N*-dimethylimidodicarbonimidic diamide) is a globally used first-line drug for the treatment of type-II diabetes and obesity. Its mechanism of action involves activation of the enzyme AMP-activated protein kinase, which inhibits gluconeogenesis in the liver, thereby reducing blood glucose (Pernicova and Korbonits, 2014). An approximate 70% of metformin is excreted unmetabolized through the human body, contributing significantly to its prevalence in aquatic habitats, impacting the native biota (Ambrosio-Albuquerque et al., 2021). Apart from metformin, its main breakdown product guanylurea (upon removal of dimethylamine) has been reported to accumulate as a dead-end product in surface waters, coastal waters and wastewater treatment plants globally at varying concentrations (Scheurer et al., 2009; Scheurer et al., 2012; Blair et al., 2013; Ghoshdastidar et al., 2015; Tao et al., 2018). For example, the concentration of metformin in German WWTP influents, effluents and surface waters was 111,800, 4,800 and 102 ng L^−1^, respectively (Trautwein et al., 2014). Whereas, in streams across the southeastern U.S., the metformin concentration was up to 16,000 ng L^−1^ (Bradley et al., 2016). WWTPs in Greece reported metformin concentrations up to 1,167 ng L^−1^ (influent) and 627 ng L^−1^ (effluent) (Kosma et al., 2015).

The complete mineralisation pathway of metformin has been described for the consortium of *Aminobacter* sp. MET and *Pseudomonas mendocina* MET (Martinez-Vaz et al., 2022). Metformin is converted to guanylurea *via* the displacement of dimethylamine (which is utilised as a carbon and nitrogen source) by *Aminobacter* sp. MET. Genome analyses of the strain further identified dimethylamine monooxygenase, which converts dimethylamine to methylamine *via* oxidation. Further, methylamine was proposed to be metabolised *via* the *N*-methyl glutamate pathway. Guanylurea was transported out of the cell by a Gdx exporter protein as it is a toxic molecule. Further, this intermediate was utilised as a sole nitrogen source by *P. mendocina* MET, which utilised all nitrogen atoms for growth. Guanylurea was converted to guanidine by the action of guanylurea hydrolase (GuuH), which was further converted to carboxyguanidine by the action of guanidine carboxylase (GC). This intermediate is metabolised to allophanate by the action of carboxyguanidine deaminase (CgdAB), which is converted to carbon-di-oxide and ammonia by the action of allophanate hydrolase (AtzF; Figure 7B). Alternatively, *P. mendocina* MET utilised metformin as a sole nitrogen source, by conversion to 1-*N*-bimethylguanide and further to biguanide. This metabolite is converted to guanylurea by a deamination reaction and is assimilated, as described (Martinez-Vaz et al., 2022).

*Aminobacter* sp. strain NyZ550 utilises metformin as a sole source of carbon, nitrogen and energy. The initial hydrolysis of metformin generates guanylurea and dimethylamine. The former accumulates as a dead-end product, while dimethylamine is utilised as a sole carbon and nitrogen source by a metabolic pathway similar to that reported in *Aminobacter* sp. MET. To further metabolise the guanylurea generated, *Pseudomonas putida* PaW340 was engineered to express guanylurea hydrolase; and both strains NyZ550 and PaW340 were co-cultured (Li et al., 2023; Figure 7B). In strain NyZ550, the genes involved in metformin metabolism were localised as three distinct clusters. Cluster I encoded the genes *tmd-tmm-dmm*DABC (Trimethylamine *N*-oxide demethylase, trimethylamine monooxygenase and dimethylamine monooxygenase), whereas cluster II encoded the genes *mgs*ABC-*gma*S-*mgd*ABCD (*N*-methylglutamate synthase, *γ*-glutamylmethylamidesynthetase and *N-*methyl glutamate dehydrogenase, respectively). Both the clusters were involved in methylamine metabolism. Whereas, cluster III encoded agmatinase and *hyp*AB (involved in loading dinickel onto agmatinase involved in metformin hydrolysis; Li et al., 2023). In *Aminobacter niigataensis* MD1 (isolated from activated sludge), the enzyme metformin hydrolase converts metformin to guanylurea and dimethylamine. The latter is utilised as the sole source of carbon and nitrogen *via* a similar route described for other isolates. Additionally, the metformin degradation gene arrangement was similar to strain NyZ550 (Chaignaud et al., 2022; Li et al., 2023).

Guanylurea metabolism in *Pseudomonas mendocina* GU proceeds *via* its hydrolytic deamination to guanidine and ammonia, which is catalysed by the enzyme guanylurea hydrolase, a novel enzyme belonging to the isochorismate hydrolase-like protein family. The bacterium utilises guanylurea but not metformin as a sole nitrogen source (Tassoulas et al., 2021). While the gene encoding *guu*H (encoding guanylurea hydrolase) was present separately on the chromosome, an ORF encoding guanidine carboxylase, carboxyguanidine deaminase (*cgd*AB) and regulatory guanidine riboswitches were clustered together. Whereas, the gene *atz*F (allophanate hydrolase) was localised adjacent to urea carboxylase and a transcriptional regulator (Tassoulas et al., 2021).

The genes *mfa*AB encoding metformin hydrolase (ureohydrolase activity) have been identified in bacteria isolated from activated sludge (Tassoulas et al., 2021). The enzyme forms an active heterocomplex that catalyses the Ni^2+^-dependent hydrolysis to guanylurea and methylamine (Li et al., 2024).

## 3 Cyanotoxins

Cyanotoxins are secondary metabolites produced by *Cyanobacteria* that are toxic to humans and other biota. Cyanotoxins are classified as per two main criteria: (1) mechanism of action, that is, hepatotoxins, neurotoxins, dermatotoxins, *etc*., and (2) chemical structure, that is, cyclic peptides (like microcystin and nodularin), alkaloids (anatoxin) or lipopolysaccharides (Ferrão-Filho and Kozlowsky-Suzuki, 2011). They are classified as emerging contaminants due to their release during extensive eutrophication/algal blooms, causing health hazards such as cancer, neuromuscular blockade, anti-acetylcholinesterase activity, anti-phosphatase activity, post synaptic cholinergic agonist activity, activation of protein kinase C, inhibition of serine/threonine protein phosphatases and inhibition of protein synthesis (Fujiki et al., 1990; Mackintosh et al., 1990; Yoshizawa et al., 1990; Codd et al., 1997; Metcalf et al., 2004; Funari and Testai, 2008; Dziga et al., 2016). Microcystins have been detected in Czech reservoir water with median and maximal concentrations of 1.5 and 18.6 μg L^−1^, respectively. Various cyanotoxins have been detected at varying concentrations in water reservoirs, fish tissue and aquatic plants in Nebraska, United States, highlighting their potential for bioaccumulation (Al-Sammak et al., 2014).

### 3.1 Microcystins and nodularin

**FIGURE 8:**
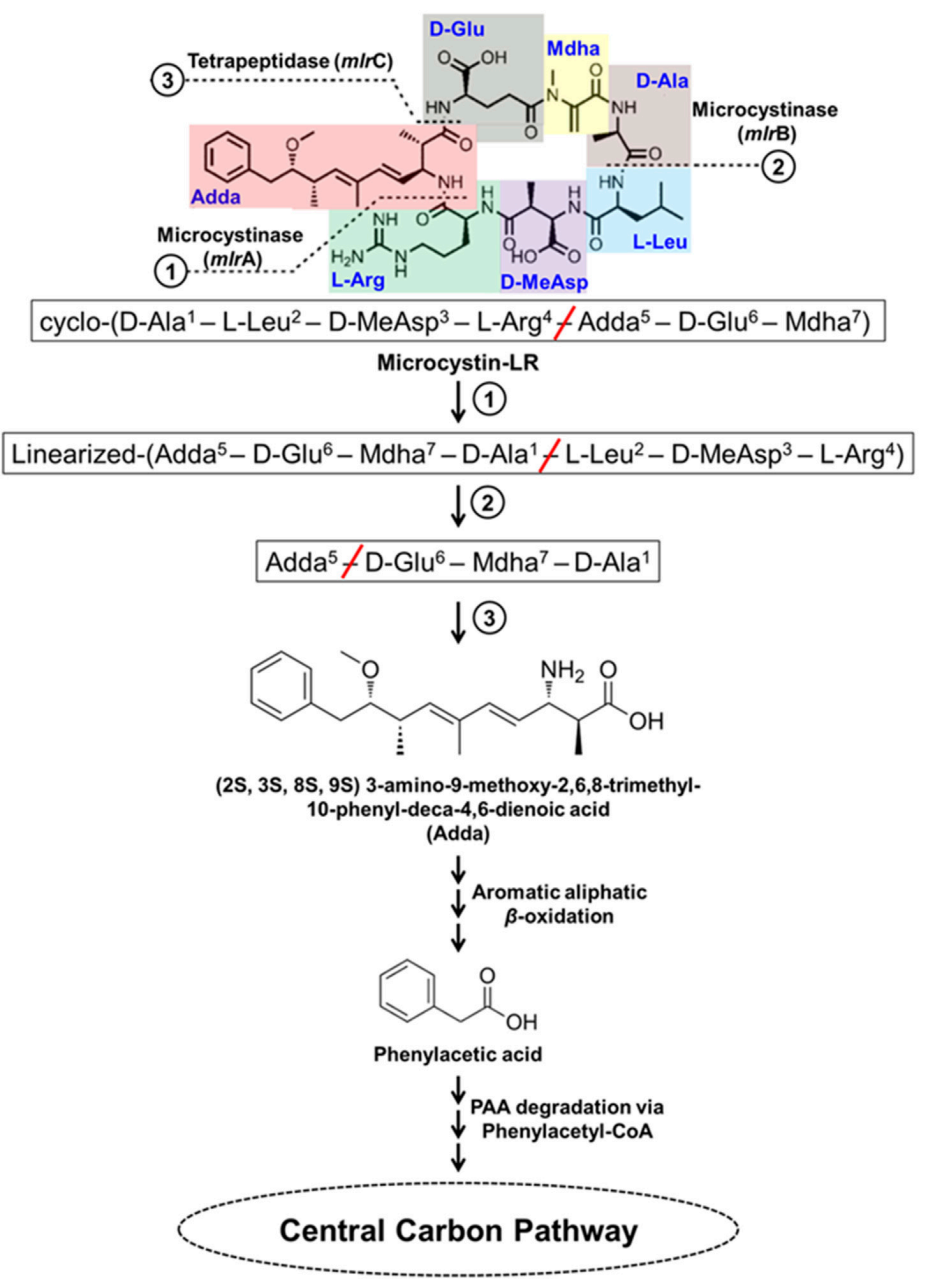
Bacterial degradation pathway of microcystin-LR. Gene encoding of the respective enzymes are indicated in parenthesis. The primary, secondary and tertiary cleavage sites (and corresponding metabolic steps) are indicated numerically in circles. Multiple arrows indicate multiple metabolic steps.

Microcystins (MCs) are the most commonly found cyanotoxins produced by several genera of *Cyanobacteria* and are the most studied. MCs are cyclic heptapeptides, comprised of cyclo-(D-Ala^1^–X^2^–D-MeAsp^3^–Z^4^–Adda^5^–D-Glu^6^–Mdha^7^) with approximately 250 identified variants (Spoof and Catherine, 2016; Yang et al., 2020). The X and Z represent variable L-amino acids (microcystins referred as MC-XZ), MeAsp is erythro-*β*-methylaspartic acid, Adda is (2S, 3S, 8S, 9S) 3-amino-9-methoxy-2,6,8-trimethyl-10-phenyl-deca-4,6-dienoic acid (*β*-amino acid), and Mdha is *N*-methyldehydroalanine (Krishnamurthy et al., 1989; Figure 8). Some examples of MC-XZ variants include MC-LR, MC-RR, MC-YR, MC-WR, MC-LY, MC-LW, MC-LF, MC-LA *etc*., where MC-LR is one of the most widely distributed and highly toxic variant.

MC-LR degradation has been detailed for *Sphingopyxis* sp. YF1. The cyclic MC-LR is first linearized by cleavage of Adda-Arg peptide bond catalyzed by microcystinase which is further acted upon by linearized microcystinase cleaving the Ala-Leu peptide bond forming a tetrapeptide containing Adda. This tetrapeptide is cleaved at Adda-Glu peptide bond by tetrapeptidase forming Adda. Adda is metabolized to form aromatic aliphatic hydrocarbon (C_20_H_26_O_4_) by the action of aminotransferase (Figure 8). The aromatic aliphatic hydrocarbon gets converted to phenylacetic acid by the microbial *β*-oxidation enzymes (fatty acid-CoA ligase, acyl-CoA dehydrogenase, enoyl-CoA hydratase, 3-hydroxyacyl-CoA dehydrogenase, and thiolase) probably in four cycles of *β*-oxidation releasing acetyl-CoA/propanoyl-CoA in each cycle. Potential intermediates formed during *β*-oxidation cycles were identified as 7-methoxy-4,6-dimethyl-8-phenyloca-2,4-dienoic acid and 2-methyl-3-methoxy-4-phenylbutyric acid. Such *β*-oxidation of aromatic aliphatic hydrocarbons has also been reported for alkylbenzenes (Sariaslani et al., 1974; Awe et al., 2008; Nhi-Cong et al., 2010; Figure 8). Further, phenylacetic acid is activated by ligating coenzyme-A catalyzed by fatty acid-CoA ligase, a phenylacetate-CoA ligase like enzyme (PAAase), in *Sphingopyxis sp.* YF1 and is proposed to be degraded *via* phenylacetyl-CoA route. The phenylacetyl-CoA is degraded to acetyl-CoA by the action of enzymes phenylacetyl-CoA epoxidase (*paa*ABCDE), 2-(1,2-epoxy-1,2-dihydrophenyl) acetyl-CoA isomerase (*paa*G), oxepin-CoA hydrolase (*paa*Z), 3-oxoadipyl-CoA thiolase (*paa*I) (Figure 8).

The genes involved in MC-LR degradation in strain YF1 include *mlr*BDAC cluster which converts MC-LR to Adda followed by aminotransferase and microbial *β*-oxidation encoding genes leading to formation of phenylacetate. Further, the *paa*I-*paa*GZ-*paa*ABCDE gene clusters encodes enzymes for degradation of phenylacetate to acetyl-CoA. The *mlr* and *paa* clusters along with genes encoding aminotransferase and *β*-oxidation enzymes were located in proximity suggesting their involvement in MC-LR degradation. The *mlr* cluster has been observed in other MC-LR degrading microbes such as *Sphingosicicella microcystinivorans* B-9 and *Novosphingobium* sp. THN1 (Jin et al., 2018; Wang J. et al., 2019; Yang et al., 2020).

Nodularin is a cyclic pentapeptide comprising of D-MeAsp^1^–L-Arg^2^–Adda^3^–D-Glu^4^–Mdhb^5^, where 1^st^–4^th^ amino acids are similar to 3^rd^–6^th^ amino acids of MC-LR and the 5^th^ Mdhb is *N*-methyldehydrobutyrine. Microcystin degrading bacteria harbouring *mlr*BDAC cluster have been observed to degrade nodularin, which is a pentapeptide possessing cleavage sites similar to MC-LR (Figure 8). As observed in *Sphingopyxis* sp. m6, during nodularin degradation *mlr* cluster was upregulated and products such as linearized nodularin and Adda were detected. This suggests nodularin degradation share similar enzymes/enzymatic steps (Yang et al., 2020; Yuan et al., 2021; Wei et al., 2023).

## 4 Plasticizers

Plasticizers are compounds used as additives to plastics to alter physical properties such as softness and flexibility. These compounds can be released into the environment during synthesis, domestic use, improper disposal or through leaching (Billings et al., 2021). Common examples of these compounds include di (2-ethylhexyl) phthalate (DEHP), dibutyl phthalate (DBP), benzyl butyl phthalate (BBP), and di-n-octyl phthalate (DnOP), which have been detected in various environmental compartments, posing significant risks to human and ecological health due to their toxicity, mutagenicity and endocrine-disrupting activity (Wang et al., 2024). In the Taihu Lake basin, China, DBP and DEHP were detected in surface waters with concentrations of 1.59 μg L^−1^ and 1.29 μg L^−1^ (mean values), respectively (Gao et al., 2019). In atmospheric samples from the North Sea, concentrations of DBP, BBP, and DEHP were found to be up to 6.6 ng L^−1^ (Xie et al., 2005). DEHP was found to occur at concentrations up to 18.5 μg L^−1^ and 0.33–97.8 μg L^−1^ in Taiwan river sediments (Yuan et al., 2002) and German surface waters (Fromme et al., 2002), respectively.

### 4.1 DEHP (Di (2-ethylhexyl) phthalate)

**FIGURE 9:**
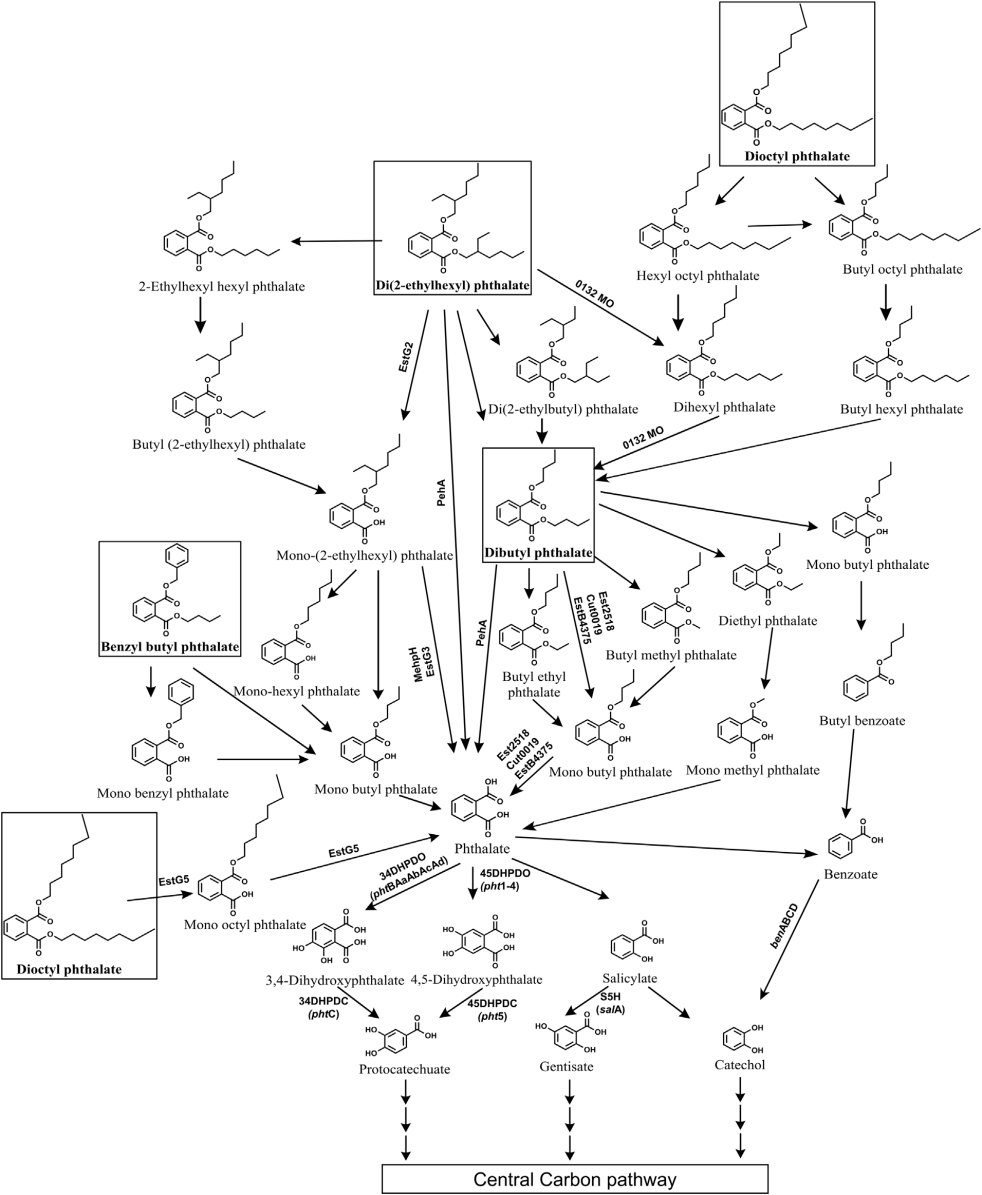
Bacterial degradation pathways of plasticizers: di (2-ethylhexyl) phthalate, dibutyl phthalate, benzyl butyl phthalate and di-n-octyl phthalate. Genes encoding respective enzymes are indicated in parenthesis. Multiple arrows indicate multiple metabolic steps. Enzyme abbreviations: 0132MO, 0132 Monooxygenase; EstG2, Esterase G2; EstG3, Esterase G3; EstG5, Esterase G5; Peh, phthalate ester hydrolase A; MehpH, mono ethylhexyl phthalate hydrolase; Est2518, Esterase 2,518; EstB4375, Esterase B4375; Cut0019, Esterase cut0019; 34DHPDO, 3,4-dihydroxyphthalate dioxygenase; 45DHPDO, 4,5-dihydroxyphthalate dioxygenase; 34DHPDC, 3,4-dihydroxyphthalate decarboxylase; 45DHPDC, 4,5-dihydroxyphthalate decarboxylase; S5H, salicylate-5-hydroxylase; BDO, benzoate dioxygenase.

Di (2-ethylhexyl) phthalate (DEHP) is the most extensively used plasticizer and is a phthalate ester composed of a phthalate backbone with two 2-ethylhexyl groups attached. It is toxic, estrogenic and a potent endocrine disrupting environmental pollutant. Several bacterial genera capable of degrading DEHP have been isolated such as *Gordonia, Rhodococcus, Mycrobacterium, Pseudomonas, Cupravidus, Burkholderia, Achromobacter, Agromyces, Microbacterium, Acinetobacter, Bacillus*, etc (Zhao et al., 2016; Xu et al., 2017; Zhang et al., 2018; Fan et al., 2018; Li et al., 2019; Wright et al., 2020; Wang et al., 2021; Chen et al., 2021a and b; Kamaraj et al., 2022; Sun et al., 2024). The initial degradation of DEHP typically occurs through two main routes: de-esterification leading to the formation of mono-alkyl esters like mono-(2-ethyhexyl) phthalate (MEHP), or stepwise beta-oxidation of alkyl side chains resulting in DBP (Figure 9). In most of bacterial strains, DEHP is hydrolysed into MEHP by esterases, which is further converted to phthalic acid, either directly or *via* mono-butyl phthalate (MBP) (Ren et al., 2016; Xu et al., 2017; Nahurira et al., 2017; Fan et al., 2018; Li et al., 2019; Lamraoui et al., 2020; Zhang H. et al., 2020; Wang et al., 2021; Kamaraj et al., 2022; Hsu et al., 2023; Kou et al., 2023; Bhattacharyya et al., 2023; Dhar et al., 2023). In other strains, the alkyl side chain of DEHP is first oxidised to DBP, which is then hydrolysed to yield phthalic acid, either directly (Zhang et al., 2021; Chen et al., 2021a; Chen et al., 2022) or through intermediates like diethyl phthalate (DEP), mono-methyl phthalate (MMP) or butyl methyl phthalate (BMP), MBP (Chen et al., 2021b; Zhang et al., 2021; Figure 9).

Some strains employ both de-esterification (*via* MEHP) and alkyl side chain oxidation (*via* DBP) routes to degrade DEHP to phthalic acid (Zhao et al., 2018; Chen et al., 2021b; Chang et al., 2022). A few strains such as *Rhodococcus pyridininvorans* DNHP-2 exhibit alternate pathways wherein DEHP undergoes conversion to 2-ethyl hexyl benzoic acid (2EHBA), which is further converted into benzoic acid (Wang et al., 2022). In *Gordonia* sp. LFF, DEHP is metabolized to phthalic acid *via* ethylhexyl hexyl phthalate (EHHP), butyl-(2-ethylhexyl) phthalate (BEHP), MEHP, mono-hexyl phthalate (MHP), and MBP (Wang et al., 2019b). In *Microbacterium* sp. DEHP1 and *Mycolibacterium phocacium* RL-HYO1, DEHP is converted to phthalic acid through intermediates di (2-ethylbutyl) phthalate (DEBP), di-n-hexyl phthalate (DnHP), DBP, and diethyl phthalate (DEP) (Ren et al., 2021; Sun et al., 2024; Figure 9).

Further, the resulting phthalic acid is converted to protocatechuate (PCA) either *via* 3,4-dihydroxyphthalate (34DHP) (Fan et al., 2018; Zhao et al., 2018; Chen et al., 2021a; Chen et al., 2021b; Bhattacharyya et al., 2023) or 4,5-dihydroxyphthalate (45DHP) (Xu et al., 2017). In few strains phthalic acid is converted to salicylate and then to gentisate or catechol (Chen et al., 2007; Ren et al., 2021). While in some bacteria, phthalic acid is converted to benzoic acid and then funneled to catechol (Chen et al., 2021a; Wang et al., 2022; Sun et al., 2024). Common intermediates like PCA, gentisate and catechol are then ring cleaved by dioxygenases and subsequently funneled into the TCA cycle (Figure 9).

### 4.2 DBP (Dibutyl phthalate)

Dibutyl phthalate (DBP) is a plasticizer that is extensively used in the production of PVC products, such as flexible plastics, vinyl flooring, and medical devices. It exhibits severe endocrine-disrupting properties as well as liver and respiratory toxicity. Several bacterial genera such as *Bacillus, Acinetobacter, Pseudomonas, Mycobacterium, Halomonas, Cupravidus, Arthrobacter, Microbacterium*, among others, have been reported to degrade DBP (Feng et al., 2018; Wright et al., 2020; Feng et al., 2021; Chen et al., 2021b; Nandi et al., 2021; Li et al., 2022; Sun et al., 2024). The degradation of DBP typically begins with its hydrolysis to mono-butyl phthalate (MBP) by esterases, either directly (Kumar and Maitra, 2016; Feng et al., 2018; Xu et al., 2022; Shariati et al., 2022; Fan et al., 2023; Sun et al., 2024) or *via* intermediates like butyl ethyl phthalate (BEP) and butyl methyl phthalate (BMP) (Feng et al., 2021; Mondal et al., 2024). Alternatively, DBP is converted to phthalic acid through intermediates such as diethyl phthalate (DEP) or dimethyl phthalate (DMP) (Sun et al., 2019; Mondal et al., 2024; Figure 9). Alternatively, in some strains, such as *Pseudomonas aeruginosa* PS1 and *Halomonas* sp. ATBC28, DBP is converted to butyl benzoate, which is further metabolized to benzoic acid (Wright et al., 2020; Du et al., 2024).

Furthermore, the resulting phthalic acid is converted to PCA *via* intermediates like 34DHP (Feng et al., 2018; Liu et al., 2020; Wright et al., 2020; Nandi et al., 2021; Chen et al., 2021b) or 45DHP (Feng et al., 2021; Du et al., 2024). In certain bacterial strains, phthalic acid is converted to benzoic acid, which undergoes decarboxylation to yield catechol. For instance, *Glutamibacter* sp 0426*, Enterobacter* DNB, and *Arthrobacter* ZJUTW convert phthalic acid to PCA *via* benzoic acid (Sun et al., 2019; Liu et al., 2020; Ren et al., 2023b; Figure 9). Similarly, in *Pseudomonas* YJB6, phthalic acid is converted to PCA *via* benzoic acid, 45DHP, and catechol (Feng et al., 2021). *Paenarthrobacter ureafaciens* PB10 converts phthalic acid into gentisate *via* 4-hydroxyphthalic acid (4HP) (Shariati et al., 2022). Common intermediates like PCA, gentisate and catechol are then subjected to ring cleavage, facilitating their entry into the tricarboxylic acid (TCA) cycle (Figure 9).

### 4.3 BBP (Benzyl butyl phthalate)

Benzyl butyl phthalate is a plasticizer that is composed of a phthalate backbone with a benzyl group and a butyl group attached to it. It is widely used in synthesis of various industrial and consumer products like PVC pipes, rubber, adhesives, cosmetics and has been reported to demonstrate reproductive and developmental toxicity, endocrine disruption, *etc*. Various bacterial genera, including *Bacillus, Acinetobacter, Arthrobacter, Gordonia*, and others, have been reported with the ability to degrade benzyl butyl phthalate (BBP) (Chatterjee and Dutta, 2003; Zhang et al., 2018; Nandi et al., 2021; Kaur et al., 2021; Fan et al., 2023). These organisms typically employ two primary pathways for BBP degradation. In the first pathway, esterases hydrolyze the alkyl side chain of BBP, yielding mono-benzyl phthalate (MBeP), which is further metabolized to phthalic acid and benzyl alcohol (Chatterjee and Dutta, 2003; Zhang et al., 2018; Kaur et al., 2021; Fan et al., 2023; Figure 9). Alternatively, in the second pathway, the aromatic side chain of BBP undergoes hydrolysis, resulting in the formation of benzyl alcohol and mono-butyl phthalate (MBP), which is then converted to phthalic acid (Chatterjee and Dutta, 2003; Zhang et al., 2018; Nandi et al., 2021; Kaur et al., 2021; Fan et al., 2023; Figure 9). Both pathways yield benzyl alcohol, which is subsequently metabolized to catechol *via* benzoic acid while the resulting phthalic acid is metabolized to PCA either *via* benzoic acid (Zhang et al., 2018) or *via* 34DHP and 3,4-dihydroxybenzoic acid (Kaur et al., 2021; Figure 9).

### 4.4 DnOP (Di-n-octyl phthalate)

Di-n-octyl phthalate (DnOP) is a type of phthalate ester commonly used as a plasticizer in various industrial applications and is composed of two octyl groups attached to a phthalate backbone. It is known to be a potent endocrine disruptor, carcinogen and immunotoxin. Several bacterial genera capable of degrading DnOP have been identified, including *Arthrobacter, Rhodococcus, Gordonia, Burkholderia, Bacillus*, among others (Wu et al., 2010; Sarkar et al., 2013; Zhang et al., 2017b; Zhang et al., 2018; Gani and Kazmi, 2018; Dhar et al., 2023). In most of bacterial strains, DnOP degradation begins with the hydrolysis by diesterase to yield mono-n-octyl phthalate (MnOP) which is subsequently converted to phthalic acid (Sarkar et al., 2013; Zhang et al., 2017b; Zhang et al., 2018; Dhar et al., 2023; Figure 9). Alternatively, in a co-culture of *Gordonia* sp. JDC-2 and *Arthrobacter* sp. JDC-32, DnOP degradation occurs through sequential *β*-oxidation of the alkyl side chain of DnOP, leading to the formation of intermediates such as hydroxyl octyl phthalate (HOP), bis(2-oxoheptyl) phthalate (BOP), DBP and DEP. DEP is then hydrolyzed to yield MMP, which is subsequently converted to phthalic acid (Wu et al., 2010; Figure 9). Similarly, in the halotolerant consortium LF, DnOP degradation was initiated by *β*-oxidation of the alkyl side chain to yield intermediates such as HOP, BOP or dihexyl phthalate (DiHP), bis(2-hydroxypropyl) phthalate (BHP), DBP, and MBP (Wang et al., 2020). The resulting phthalic acid was converted to PCA either *via* dihydroxyphthalates (Zhang et al., 2018; Dhar et al., 2023) or benzoic acid (Zhang et al., 2018; Figure 9).

## 5 Pesticides

Pesticides belonging to the class of aniline derivatives, carbamates, chlorophenoxy compounds, chloroacetanilides, organochlorines, organophosphates, triazines, and neonicotinoids occur as CECs and pose major concern due to their high persistence, leachability, bioaccumulative nature and potential toxicity (Salimi et al., 2017; Khezami et al., 2024). These compounds are used in agricultural as well as non-agricultural settings for protection of crops against insects, fungi, nematodes, *etc*., as well as for the control of unwanted herbs and have been found to occur in various ecological compartments. For example, glyphosate was found to occur at 0.21–1.3 mg kg^−1^ soil in medlar planting site in Golmud, China (Jing et al., 2021). Imidacloprid and carbendazim have been detected in dust samples in China with concentrations of 25.8 ng g^−1^ and 35.8 ng g^−1^, respectively (Wang A. et al., 2019). In Italian house dust, imidacloprid and carbendazim were found to occur at concentrations between 1.6 and 39 μg g^−1^ and 0.08–4.9 μg g^−1^, respectively (Salis et al., 2017). The persistence and slow rate of natural attenuation of pesticides has led to various health and environmental issues. Majority of these compounds are mutagenic, endocrine disrupting, carcinogenic and are known to cause environmental hazards, compromised soil health and toxicity to biota, including humans (Choi et al., 2004).

### 5.1 Imidacloprid

Neonicotinoids are a recent class of pesticides consisting of thiacloprid, acetamiprid, imadacloprid, clothianidin, *etc*., used for crop protection, horticulture, and flea control. Imidacloprid is the most commonly used insecticide of the neonicotinoid group and is recognized as a contaminant of emerging concern (Selvam and Srinivasan, 2019; Petkovic Didovic et al., 2022). Low bioavailability of imidacloprid results in slow rate of natural attenuation and leads to longer half-life (∼997 days) in soil. Extensive use of imidacloprid exerts adverse effects on non-target species like fish, bees, earthworm, mice, human, *etc*. (Phugare et al., 2013; Pang et al., 2020).

**FIGURE 10:**
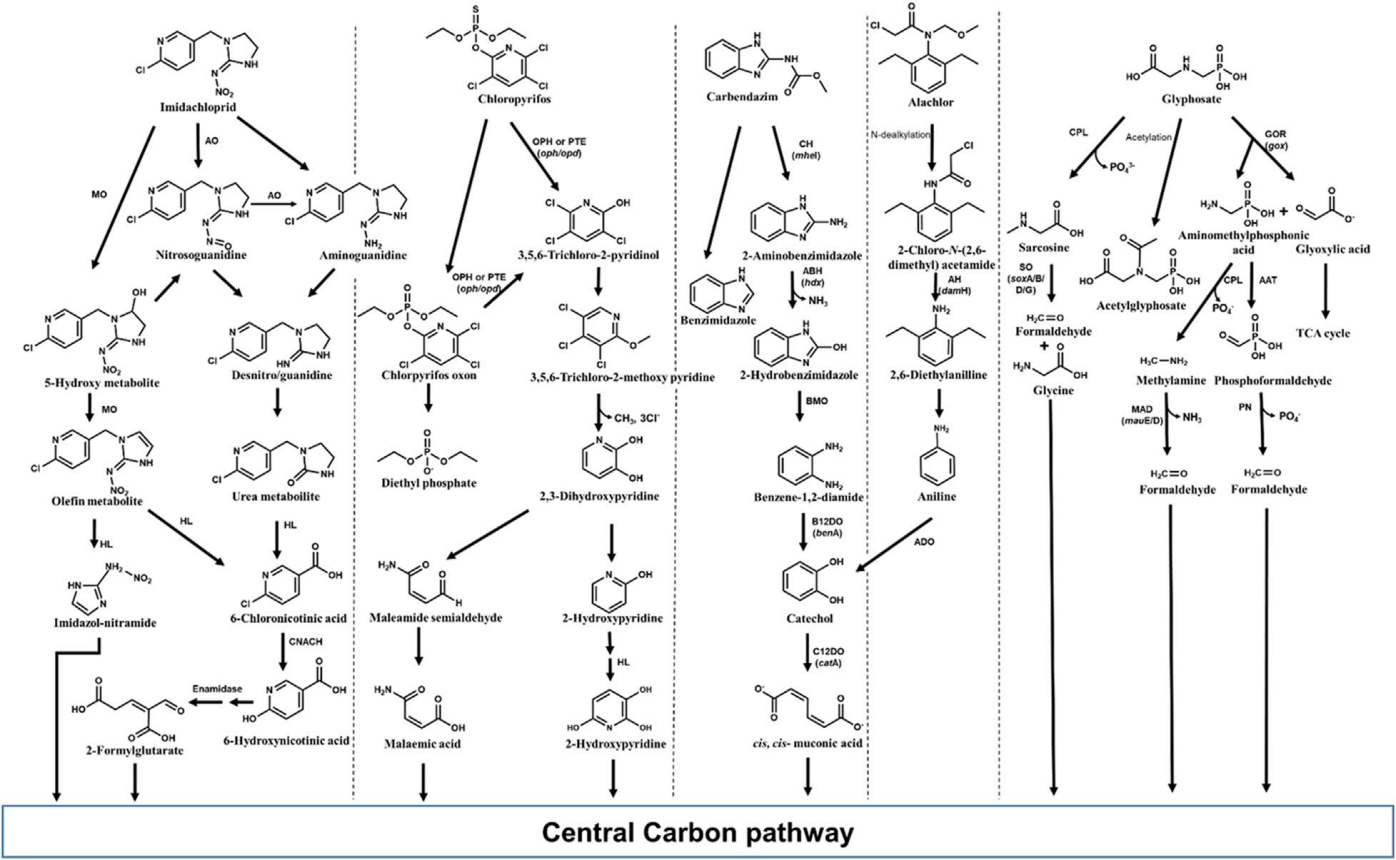
Metabolic pathways for degradation of various pesticides of emerging concern in bacteria. Genes encoding respective enzymes are indicated in parenthesis. Enzyme abbreviations: CPL, C-P lyase; GOR, Glyphosate oxydoreductase; ATT, AMPA aminotransferase; SO, Sarcosine oxidase; PN, Phosphonatase; MAD, methyl amine dehydrogenase; ADO, Aniline dioxygenase; C12DO, Catechol-1,2-dioxygenase; AH, amide hydrolase; CH, Carbendazim hydrolase; ABH, 2-Aminobenzimidazole hydrolase; BMO, 2-Hydrobenzimidazole monooxygenase; B12DO, Benzoate-1,2-dioxygenase; MO, monooxygenase; AO, Aldehyde oxidase; HL, Hydrolase; CNACH, 6-Chloronicotinic acid chlorohydrolase; OPH, Organophosphate hydrolase; PTE, Phosphotriesterase.

Various bacteria including *Pseudomonas, Bacillus, Klebsiella, Mycobacterium, etc*., have been reported to degrade imidacloprid with various possible pathways. Among the reported routes, oxidation and nitro-reduction are two major microbial biodegradation pathways of imidacloprid (Pandey et al., 2009; Phugare et al., 2013; Pang et al., 2020; Zhang X. et al., 2023). 6-Chloronicotinic acid (6-CNA), olefinic cyclic nitroguanidine, cyclic urea, cyclic guanidine, nitroso, and nitro derivatives are major metabolites of imidacloprid nitro-reduction detected in soil and water samples. Under microaerophilic conditions, an aldehyde oxidase converts the ‘magic nitro’ group of imidacloprid to a nitrosoguanidine metabolite. The imidacloprid and/or formed product i.e., nitrosoguanidine is degraded through a more toxic nitroguanidine intermediate which is further converted into non-toxic urea metabolites (Pandey et al., 2009; Figure 10). In some microorganisms, imidacloprid is cleaved to 6-CNA by the formation of nitrosoguanidine and oxidative cleavage of guanidine residue (Phugare et al., 2013). Alternatively, imidacloprid is converted to 6-CNA *via* formation of 5-hydroxy and olefin metabolites by subsequent hydroxylation and dehydrogenation. 6-CNA is eventually converted to CO_2_ *via* 6-hydroxynicotinic acid (Sharma et al., 2014; Figure 10).

### 5.2 Chlorpyrifos

Chlorpyrifos, [*O*,*O*-diethyl *O*-(3,5,6-trichloro-2-pyridinyl)-phosphorothioate], is a broad-spectrum, chlorinated organophosphate insecticide, acaricide and miticide used to control foliage- and soil-borne insect pests on a variety of food and feed crops (Lara-Moreno et al., 2022; Bosu et al., 2024). The major health issues caused by chlorpyrifos include respiratory, immunological, reproductive, and neurological disorders in humans (Anwar et al., 2009). Although potential health risks have led to the ban of chlorpyrifos in many countries, it has been approved for limited use in densely populated countries like India, China and Bangladesh (Lara-Moreno et al., 2022). In Mexico, for example, extensive use of chlorpyrifos from 2012 to 2020 resulted in contamination of waterbodies (estuaries, drains and artesian wells) with an average concentration of 4,614 ng L^−1^ of chlorpyrifos (Ruiz-Arias et al., 2023). Chlorpyrifos has an average half-life of around 60–120 days in the soil, depending upon climate and soil stability (Anwar et al., 2009; Bosu et al., 2024). Various microorganisms belonging to the genera *Arthrobacter, Enterobacter, Xanthomonas, Streptomyces, Stenotrophomonas, Sphingomonas, Bacillus, Synechocystis, Pseudomonas, Actinobacteria,* and *Klebsiella* have been identified as potential chlorpyrifos degraders (Singh, 2009; Ambreen and Yasmin, 2021).

Various microorganisms are known to produce metal-dependant enzymes (hydrolases) such as organophosphorus hydrolase, phosphotriesterase (PTE), methyl parathion hydrolase and organophosphorus acid anhydrolase (OPAA) involved in chlorpyrifos bioremediation (John and Shaike, 2015; Bosu et al., 2024). Organophosphorus hydrolase effectively cleaves P–O bond in the phosphotriesters, and P–S linkage in the phosphothiolesters, yielding two major metabolites, 3,5,6-trichloro-2-pyridinol (TCP) and diethylphosphate (DETP) from chlorpyrifos. Other minor metabolites such as desethyl chlorpyrifos, chlorpyrifos oxon, desethyl chlorpyrifos oxon, and 3,5,6-trichloro-2-methoxypyridine (TMP) are also produced. Chlorpyrifos oxon, the oxidized form of chlorpyrifos, is further hydrolyzed either enzymatically or spontaneously to form diethylphosphate and TCP. The TCP can be further degraded to TMP and CO_2_ (Racke, 1993; John and Shaike, 2015; Figure 10).

### 5.3 Carbendazim

Carbendazim (methyl *N*-(1*H*-benzimidazol-2-yl) carbamate) is a systemic broad-spectrum fungicide, which is also formed as a degradation product of thiophanate-methyl and benomyl fungicides (Mazellier et al., 2003; Fang et al., 2010). Carbendazim is used worldwide as pre- and post-harvest treatment to control the Ascomycetes, Fungi imperfecti and Basidiomycetes fungal diseases on various vegetables, fruits and several other plants. Carbendazim was found to be toxic to various animals and could induce reproductive, developmental, endocrine and haematological toxicity (Rama et al., 2014; Zhou et al., 2023). Many microorganisms, predominantly bacteria such as *Rhodococcus, Nocardioides, Mycobacterium, Pseudomonas*, *etc*., have been reported to metabolize carbendazim (Singh et al., 2016; Zhou et al., 2023). Among reported microbes, few bacterial strains have been found to be efficient degraders of carbendazim. For example, *Rhodococcus* sp. D-1 isolated from contaminated farmland in China, could degrade 98.20% of 200 ppm carbendazim within 5 days (Bai et al., 2017). In most organisms, carbendazim degradation is initiated by its hydrolysis to 2-aminobenzimidazole (2-AB) and further, 2-hydroxybenzimidazole (2-HB) (Wang et al., 2010; Arya et al., 2015; Figure 10). Subsequently, 2-HB is converted to catechol *via* the formation of benzene-1,2-diamine and further channelled into TCA cycle (Singh et al., 2016; Figure 10).

### 5.4 Alachlor

Chloroacetanilide herbicides such as alachlor, metolachlor, and acetochlor are primary herbicides, and more than 50 million kg has been used annually in the United States (Gan et al., 2002). These herbicides are highly soluble in water and persist in soil, with residues or metabolites being detected in surface and ground water (Potter and Carpenter, 1995; Tian et al., 2021). For example, alachlor was detected in groundwaters in the United States at concentration 0.1–16.6 μg L^−1^, exceeding the U.S. Environmental Protection Agency (US-EPA) maximum contaminant level criteria of 2 μg L^−1^ (WHO, 2017).

Alachlor [2-chloro-*N*-(2,6-diethylphenyl)-*N*-(methoxymethyl]acetamide] is one of the majorly used chloroacetanilide applied as a selective pre- and post-emergent herbicide to control weeds in soybeans, peanuts, and corn crops. *C*-dealkylation of other chloroacetanilide herbicides like butachlor leads to formation of alachlor. Alachlor has been categorised as a human carcinogen and has been reported to mimic 17*β*-estradiol, thereby acting as an endocrine-disruptor (Lee and Kim, 2022). Microbes including various genera of bacteria like *Paracoccus, Rhodococcus, Pseudomonas, Acinetobacter, Streptomyces, etc.*, and few fungi like *Paecilomyces* have been reported to degrade alachlor (Słaba et al., 2013; Lee and Kim, 2022; Chen et al., 2023). Alachlor is converted to 2-chloro-*N*-(2,6-diethylphenyl) acetamide (CDEPA) by *N*-dealkylation (Zhang et al., 2011; Figure 10). Various hydrolases/reductases have been reported to be involved in *N*-dealkylation of chloroacetanilides. For example, enzyme ChlH from *Rhodococcus* sp. B1 and enzymes CndB1, CndB2, and CndC1 from *Sphingomonas* sp. DC-6 have been reported to catalyse the *N*-dealkylation of alachlor as well as other chloroacetamide like acetochlor, butachlor, and pertilachlor (Chen et al., 2023; Figure 10). Subsequently, CDEPA is transformed to 2,6-diethylanilline (DEA) with the help of enzyme amidase (CmeH) or amide hydrolase (DamH). DEA is further converted to aniline which is then acted upon by aniline dioxygenase to form catechol. The formed catechol is then oxidized through an *ortho*-cleavage pathway to *cis, cis*-muconic acid and funnelled into TCA cycle (Zhang et al., 2011; Kim et al., 2013; Gao et al., 2015; Figure 10).

### 5.5 Glyphosate

Glyphosate is a low-molecular-weight phosphonate (non-specific organophosphate herbicide) with high aqueous solubility and mobility, which leads to rapid leaching of this compound into soil, causing contamination of water bodies. Glyphosate has been reported to cause toxicity to bacteria as well as multicellular organisms like non-target crop plants, crustaceans, molluscs and chordates including humans (cytotoxicity and genotoxicity) (Zhan et al., 2018; Singh et al., 2020). Various strains of *Achromobacter, Agrobacterium, Pseudomonas, Ochrobactrum*, *etc*., have been previously isolated from contaminated sites which can utilize glyphosate as growth substrate (Zhao et al., 2015; Zhan et al., 2018; Feng D. et al., 2020). The primary degradation products of glyphosate include aminomethylphosphonic acid (AMPA) and sarcosine, which are reported to be more toxic than the parent compound (Zhan et al., 2018; Lozano and Pizarro, 2024; Figure 10). C-P lyase removes phosphate group from glyphosate yielding sarcosine, which is cleaved by sarcosine oxidase (encoded by 7 *sox* genes) into glycine and formaldehyde. Both these intermediates are funnelled into microbial metabolism and biosynthetic pathways (González-Valenzuela and Dussán, 2018; Diaz-Soto et al., 2024; Figure 10). In many microorganisms, glyphosate is converted to AMPA and glyoxylate by the action of glyphosate oxidoreductase. Further, glyoxylate is metabolized to TCA cycle, whereas AMPA is either acted upon by C-P lyase to produce methylamine or by an aminotransferase to form formylphosphonate. Both the intermediates i.e., methylamine and formylphosphonate are cleaved to formaldehyde, which is used by microbes for biosynthesis (Sviridov et al., 2015; Zhao et al., 2015; Singh et al., 2019; Figure 10).

### 5.6 Application of OMICS and metabolic engineering to CEC degradation

The application of various omics approaches to CEC degradation can provide possible systemic-level insights into the metabolic pathways and associated regulatory mechanisms. Genomics aids in identifying key genes encoding degradative enzymes and/or the evolutionary trajectory. For example, in strain *Pseudomonas* sp. C5pp, the draft genome analysis revealed the presence of three gene clusters on a single contig (Supercontig-A) involved in complete Carbaryl degradation. The genome analysis further suggested acquisition of genes by horizontal gene transfer events (Trivedi et al., 2016). Genomics in conjunction with transcriptomics and proteomics aids in identifying up/downregulation of genes/proteins under target conditions. For example, in *P. bharatica* CSV86^T^, the transcription analysis showed the induction of target genes involved in benzoate (*ben*E, *ben*K) and glucose (*gbp*, *opr*B, *glc*G) utilisation (Choudhary et al., 2017). Further proteomic analysis aided in identification of Gbp and OprB as glucose binding protein and carbohydrate specific porin, respectively which are induced when the culture is grown on glucose (Basu et al., 2007; Shrivastava et al., 2011). In *Methylorubrum* sp. ZY-1, the integrated transcriptomic and metabolomic analyses aided in revealing degradation of pentachlorodiphenyl (PCB 118) and underlying molecular mechanisms (Wu et al., 2024). Table 2 provides a comprehensive review of the application of omics in CEC biodegradation for pharmaceuticals, cyanotoxins, plasticizers and pesticides, which are also described further.

**TABLE 2:** Application of various OMICS techniques for degradation of contaminants of emerging concern.

Organism	Growth/Degradation substrate	Omics technique used	Key findings	Reference
*Patulibacter* sp. I11	Ibuprofen	Proteomics	Enoyl-CoA hydratase/isomerase and acyl-CoA synthetase enzymes are upregulated and involved in ibuprofen degradation. ABC transporter upregulated and probably involved in ibuprofen uptake.	Almeida et al. (2013)
*Comamonas*, *Pseudomonas*	Testosterone	Metagenomics	*Comamonas* and *Pseudomonas* are involved in testosterone degradation in sludge samples. *meta*-cleavage dioxygenase gene *tes*B is upregulated and involved in testosterone degradation.	Chen et al. (2016)
*Burkholderia* sp. ABC02, *Pseudomonas* sp. ABC07, *Pandoraea* spp. ABC08 , ABC10	Penicillin, Benzylpenicilloic acid, Phenylacetic acid	Comparative transcriptomics	*bla* (*beta*-lactamase) and *put* (amidase) genes upregulated in presence of penicillin and benzylpenicilloic acid, but not phenylacetic acid. *paa* (phenylacetic acid) operon was upregulated in response to all three intermediates (penicillin, benzylpenicilloic acid and phenylacetic acid), revealing metabolic architecture of penicillin degradation.	Crofts et al. (2018)
*Arthrobacter* sp. D2, *Pimelobacter* sp. LG209	Sulphadiazine	Metagenomics	*Arthrobacter* and *Pimelobacter* are dominant members of a sulphadiazine-degrading consortia. *Arthrobacter* sp. D2 converted sulphadiazine to 2-aminopyrimidine, *Pimelobacter* sp. LG209 mineralised this intermediate.	Deng et al. (2018)
*Lysinibacillus sphaericus*	Glyphosate	Genomics	Sarcosine oxidase is upregulated and involved in glyphosate degradation	González-Valenzuela and Dussán (2018)
*Arthrobacter* sp. ZJUTW	DBP	Genomics, transcriptomics	Alpha-ketoglutarate transporter, chaperones, MFS transporters, flavin-dependent oxidoreductases, and NADPH-dependent FMN reductase genes are upregulated in presence of DBP. Identification of *peh*A gene encoding a DBP-hydrolyzing esterase. Identification of *pht* cluster responsible for converting phthalic acid to protocatechuate located on a plasmid. Identification of *pca* gene clusters responsible for converting PCA to TCA cycle intermediates located on the chromosome.	Liu et al. (2020)
*Halomonas* sp. ATBC28 , *Mycobacterium* sp. DBP42	DBP and DEHP	Genomics, proteomics, metabolomics	Upregulation of active membrane transporters (TRAP transporters) and a membrane-linked OmpA-like protein (strain ATBC28) upon plasticizer exposure. Identification of key esterases for DBP hydrolysis (cutinase 0019 in strain DBP42, esterase 4,375 in strain ATBC28) and *pht-pca/ben-cat* gene clusters. DBP degradation involves sequential removal of the ester-bound side chains, producing phthalate and butanol, while DEHP follows a sequential shortening of the side chains.	Wright et al. (2020)
*Serratia nematodiphila* MB307	Ibuprofen	Proteomics	13 proteins including Fe-S cluster scaffold-like protein (*isc*U), autoinducer-2 modifying protein (*lsr*G) and peptidylprolyl isomerase upregulated in presence of ibuprofen. IscU, LsrG and peptidylprolyl isomerase play a role in stress tolerance to ibuprofen.	Basharat et al. (2022)
Bacterial consortium CL	Chloramphenicol	Metagenomics, meta-transcriptomics, proteomics	*Sphingomonas*, *Caballeronia*, *Cupriavidus* and *Pigmentiphaga* are major players in chloramphenicol degradation. CapO (glucose-methanol-choline oxidoreductase), nitroreductase, chloramphenicol acetyltransferase are upregulated and involved in chloramphenicol degradation.	Zhang et al. (2022)
*Microbacterium* sp. C448	Sulphamethazine	Transcriptomics, proteomics	The transcript and protein levels of the degradation enzymes monooxygenase SadA and flavin reductase SadC exhibited increase in presence of sulphamethazine. The upregulation of the enzyme RidA (reactive intermediate deaminase A) suggested its potential role in deamination of 2-aminophenol. The putative sulphate exporter family protein showed upregulation in presence of sulphamethazine.	Paris et al. (2023)
*Actinobacteria*, *Proteobacteria*	Oestrogen	Metagenomics and comparative genomics	*Actinobacteria* and *Proteobacteria* are major players in oestrogen biodegradation distributed in aquatic ecosystems. *aed*J and *edc*C are biomarkers for oestrogen degradation in *Actinobacteria* and *Proteobacteria*.	Hsiao et al. (2023)
*Gordonia* sp. GONU	DOP and DEHP	Genomics, Proteomics	Identification of key esterases involved in initial hydrolysis of DOP and DEHP to phthalate. Identification of phthalate degradation *pht* and protocatechuate degradation *pca* gene clusters. The esterases EstG5 and EstG3 are specifically expressed to hydrolyse DnOP to phthalate, whereas EstG2 and EstG3 are specifically expressed to metabolise of DEHP to phthalate *via* MEHP.	Dhar et al. (2023)
*Shingopyxis* sp. YF1	Microcystin-LR	Genomics, Transcriptomics, metabolomics	Identification of *mlr* and *paa* cluster as well as fatty-acid *β*-oxidation genes and corresponding enzymes involved in complete degradation of MC-LR.	Yang et al., 2020; Wei et al., 2023
*Paracoccus* sp. APAP_BH8	Acetaminophen	Genomics, proteomics, metabolomics	M20 aminoacylase (amidohydrolase), guanine deaminase GuaD, 4-hydroxybenzoate-3-monoxygenase PobA and 4-hydroxyphenyl pyruvate dioxygenase HppD are upregulated and involved in acetaminophen degradation. 4-aminophenol, hydroquinone and 3-hydroxy *cis*-*cis* muconate are acetaminophen degradation pathway intermediates.	Pandey et al. (2024)
*Microbacterium* DEHP1	DBP and DEHP	Genomics, metabolomics	Two key esterases – est 2518 and 0132monooxygenase identified involved in the degradation of DBP and DEHP. Three operons (*ben*, *cat*, *pca*) involved in the degradation of DBP and DEHP. DBP could be hydrolysed by esterase 2,518 to yield mono-butyl phthalate and subsequently phthalic acid whereas DEHP may be converted to di-n-hexyl phthalate and then DBP by monooxygenase 0132.	Sun et al. (2024)
*Burkholderia cenocepacia* CEIB S5-2	Glyphosate	Genomics	Degradation of glyphosate proceeds *via* both sarcosine and AMPA pathway.	Díaz Soto et al. (2024)
*Pseudomonas aeruginosa* PS1	DBP	Genomics, transcriptomics	Identification of 66 key genes involved in a unique DBP metabolism pathway.	Du et al. (2024)

### 5.7 Pharmaceuticals

Comparative transcriptomic analyses of four penicillin degrading strains revealed upregulation of genes encoding beta-lactamase (*bla*), penicillin amidase (*put*) and phenylacetic acid degradation enzymes (*paa*) in penicillin grown cells (as compared to alternative carbon source grown cells). In *Pseudomonas* sp. strain ABC07, the *put* operon, encoding four open reading frames: a *beta*-lactamase, a major facilitator family importer and two amidases (*put*1 and *put*2) was found to be upregulated in presence of penicillin and benzylpenicilloic acid, but not phenylacetic acid. Whereas, the *paa* operon was responsive to all three intermediates (penicillin, benzylpenicilloic acid and phenylacetic acid). Therefore, these analyses indicated the metabolic architecture of penicillin degradation in strain ABC07 (Crofts et al., 2018). A combination of metagenomic and cultivation-based techniques identified *Arthrobacter* and *Pimelobacter* as the dominant members of a sulphadiazine-degrading consortia as well as their individual roles in degradation. While *Arthrobacter* sp. D2 converted sulphadiazine to 2-aminopyrimidine, *Pimelobacter* sp. LG209 mineralised this intermediate (Deng et al., 2018). Exposure of *Microbacterium* sp. C448 to therapeutic and sub-therapeutic doses of sulphamethazine was assessed using transcriptomic and proteomic analyses. The transcript and protein levels of the degradation enzymes monooxygenase SadA and flavin reductase SadC exhibited increase in presence of sulphamethazine. Further, the upregulation of the enzyme RidA (reactive intermediate deaminase A) suggested its potential role in deamination of 2-aminophenol. Additionally, the putative sulphate exporter family protein showed upregulation in presence of sulphamethazine (Paris et al., 2023). An integrated multi-omics approach revealed the chloramphenicol biotransformation pathway, genes, proteins/enzymes and community structure/interactions of the activated sludge enriched consortium CL. The metagenomic analysis revealed *Sphingomonas*, *Caballeronia*, *Cupriavidus* and *Pigmentiphaga* as the major players in chloramphenicol degradation. Further, metatranscriptomic analysis revealed upregulation of specific detoxification and metabolic pathway genes such as *cap*O, which encodes a glucose-methanol-choline oxidoreductase responsible for oxidation of C_3_-OH group of chloramphenicol. The proteomic analysis validated the metatranscriptomic data and the functionality of the identified enzymes such as CapO, nitroreductase and chloramphenicol acetyltransferase (Zhang et al., 2022).

The genomic and proteomic analysis of sulphamethoxazole-degrading *Pseudomonas silesiensis* F6a revealed six key degradation genes, *deo*C (2-deoxyribose 5-phosphate aldolase), *nar*I (nitrate reductase), *lux*S (S-ribosyl homocysteine lyase), *nuo*H (NADH quinone oxidoreductase), gene 0655 (F420 dependent oxidoreductase) and gene 4,650 (amidohydrolase) involved in C-S bond cleavage, S-N bond hydrolysis and isoxazole ring-cleavage (Liu et al., 2022).

Metabolomic analyses of *Sphingobacterium mizutaii* S121 revealed the products of tetracycline biodegradation by the strain and the stress response mechanisms involved. Based on the analyses, two biodegradation pathways involving demethylation and one hydrolysis pathway were proposed. The levels of indole, glutamic acid and FAD, involved in regulating the activity of efflux proteins and degradation enzymes, were upregulated. Further, intracellular levels of nucleotides and amino acids were significantly increased to repair DNA/RNA and protein in response to tetracycline stress. The levels of antioxidants such as taurine and protoporphyrin IX also increased in response to the generation of reactive oxygen species due to enhanced aerobic metabolism. Under tetracycline stress, strain S121 required increased nutrients from the extracellular environment, due to which the levels of the metabolite *N*-palmitoyl sphingomyelin and phosphoethanolamine decreased significantly, enhancing membrane fluidity (Tan H. et al., 2022).

A metagenomic analysis of sewage samples incubated with testosterone indicated the genera *Comamonas* and *Pseudomonas* to be major players in degradation. Further, the *meta*-cleavage dioxygenase gene *tes*B was identified and exhibited a significant increase after 48 h of incubation (Chen et al., 2016). A combination of metagenomic analyses and comparative genomics revealed *Actinobacteria* and *Proteobacteria* as major players in oestrogen biodegradation distributed in aquatic ecosystems. Further, *aed*J and *edc*C were identified as biomarkers for oestrogen degradation in *Actinobacteria* and *Proteobacteria*, respectively, with potential application for environmental detection (Hsiao et al., 2023).

Quantitative proteomics of *Patulibacter* sp. I11 in absence or presence of ibuprofen revealed likely proteins involved in degradation. In presence of ibuprofen, various proteins involved in aromatic degradation such as enoyl-CoA hydratase/isomerase, acyl-CoA synthetase, Rieske (2Fe-2S) domain containing were upregulated. Additionally, other proteins such as ABC transporter (probably involved in ibuprofen uptake), putative lyase, stress response protein and AMP-forming synthetase were also upregulated (Almeida et al., 2013). The stress response of *Serratia nematodiphila* sp. MB307 to the presence of ibuprofen was investigated using differential proteomics. Thirteen proteins were upregulated and 29 proteins were downregulated in response to ibuprofen stress. Among the upregulated proteins, Fe-S cluster scaffold-like protein IscU, autoinducer-2 modifying protein LsrG and peptidylprolyl isomerase have been implicated for their role in stress tolerance. Overall, the analyses highlighted the multifaceted stress response of strain MB307, involving a balance between protein synthesis, DNA replication, and energy production (Basharat et al., 2022).

The genomic-proteomic-metabolomic analyses of *Paracoccus* sp. APAP_BH8 elucidated the genes, enzymes and metabolic pathway of acetaminophen degradation in the strain. The proteome analysis revealed the upregulation of M20 aminoacylase (amidohydrolase), guanine deaminase GuaD, 4-hydroxybenzoate-3-monoxygenase PobA and 4-hydroxyphenyl pyruvate dioxygenase HppD in presence of acetaminophen. Molecular docking studies of these enzymes with their respective substrates validated the functionality of these enzymes. Further, the metabolomic analysis revealed 4-aminophenol, hydroquinone and 3-hydroxy *cis, cis*-muconate as degradation pathway intermediates (Pandey et al., 2024).

Genomic and comparative transcriptome analyses of *Aminobacter* sp. Strain NyZ550 revealed upregulation of the genes *dmm* (dimethylamine monooxygenase), *gmas* (γ-glutamylmethylamide synthetase), *mgs* (*N*-methylglutamate synthase) and *mgd* (*N*-methylglutamate dehydrogenase) while growing on metformin, indicating the metabolism of dimethylamine. Further, the serine cycle and formate-tetrahydrofolate catabolic genes also exhibited upregulation. Importantly, the gene encoding agmatinase (putative metformin hydrolase) exhibited upregulation, highlighting its role in degradation (Li et al., 2023).

### 5.8 Cyanotoxins

In *Sphingopyxis* sp. YF1, genomic analysis revealed the presence of MC degrading cluster *mlr*BDAC. The MC-LR degradation products such as linearized MC-LR, tetrapeptide, Adda and its degradation intermediates, and phenylacetic acid were detected using UPLC and UPLC-ESI-MS. Further, transcriptomics and qRT-PCR analyses suggested the upregulation of *mlr* cluster, fatty acid *β*-oxidation genes and *paa* cluster during MC-LR degradation. Metabolomics study showed enrichment of metabolites in pantothenate and CoA biosynthesis as well as fatty-acid degradation indicating involvement of fatty acid *β*-oxidation in MC-LR degradation (Wei et al., 2023). In *Sphingopyxis* sp. m6, gene specific qPCR suggested involvement of *mlr* cluster in nodularin degradation and the respective enzymatic steps were identified by metabolite analysis using total ion chromatogram (Yuan et al., 2021).

### 5.9 Plasticizers

In *Gordonia* sp. GONU, genome sequencing aided in identification of key esterases involved in initial hydrolysis DOP and DEHP to phthalate as well as *pht* gene clusters (responsible for conversion of phthalate to protocatechuate) and *pca* gene clusters (responsible for conversion of protocatechuate to TCA cycle intermediates). Substrate dependent gene expression profile by qRT-PCR and protein profiling by LC-ESI-MS/MS revealed that esterases EstG5 and EstG3 are specifically expressed to hydrolyse DnOP to PA, whereas EstG2 and EstG3 are specifically expressed to metabolise DEHP to PA *via* MEHP (Dhar et al., 2023).

Genome sequencing of *Microbacterium* sp. DEHP1 identified two key esterases-*est*258 and monooxygenase 0132 as well as three operons (*ben, cat, pca*) involved in the degradation of DBP and DEHP. Genome mining and metabolite identification by GC-MS suggested that DBP could be hydrolyzed by esterase 2,518 to yield mono-butyl phthalate (MBP) and subsequently phthalic acid (PA) whereas DEHP may be converted to di-n-hexyl phthalate (DnHP) and then DBP by monooxygenase 0132. Further, metabolic profiling using UHPLC-QTOF/MS revealed that under DEHP/DBP stress, strain DEHP1 cells showed increased levels of valine (which induces production of osmoregulatory substances), glycerophospholipids (major component of cell membrane), glutathione/protoanemonin (antioxidants), and proline (key player to preserve cellular glutathione redox status by activating signaling pathway). Notably, levels of organic substances like levan and naringenin 4′-*O*-alpha-L-rhamnopyranoside decreased in response to DEHP stress (Sun et al., 2024).

A combined genomic and transcriptomic approach identified 66 key genes involved in two different mono-butyl phthalate-catabolism steps in *Pseudomonas aeruginosa* PS1. In addition to the genes encoding the metabolic pathway enzymes, most differentially expressed genes in *Pseudomonas aeruginosa* PS1 under DBP stress were those encoding for ABC transporters, two-component systems, biofilm formation, quorum sensing and chemotaxis (Du et al., 2024).

The genome sequencing of DBP degrading *Arthrobacter* sp. ZJUTW identified the presence of *peh*A gene encoding a DBP-hydrolyzing esterase and *pht* gene cluster responsible for converting phthalic acid to protocatechuate located on a plasmid, and *pca* gene clusters responsible for converting PCA to TCA cycle intermediates located on the chromosome. Additionally, transcriptomic analysis by RNA-seq showed the upregulation of genes encoding an alpha-ketoglutarate transporter (important for cell wall synthesis), chaperones, MFS transporters (important for DBP efflux), flavin-dependent oxidoreductases, and NADPH-dependent FMN reductase genes (Liu et al., 2020).

Proteogenomic and metabolomic analysis of *Halomonas* sp. ATBC28 and *Mycobacterium* sp. DBP42 identified key esterases for DBP hydrolysis (cutinase 0019 in strain DBP42, esterase 4,375 in strain ATBC28) and *pht-pca/ben-cat* gene clusters. Metabolite analysis revealed that DBP degradation involves sequential removal of the ester-bound side chains, producing phthalate and butanol, while DEHP follows a sequential shortening of the side chains. Further, in strain ATBC28, active membrane transporters (TRAP transporters 0264 and 0631) and a membrane-linked OmpA-like protein (3,348) were upregulated, potentially for detoxification and biosurfactant production, respectively (Wright et al., 2020).

### 5.10 Pesticides

Proteomics and metabolomics provided an enhanced understanding of alachlor biodegradation by *P. marquandii*. Metabolomics (by LC-MS/MS) suggested that presence of alachlor reduced the culture growth and glucose consumption rates and increased the formation of supplementary materials (UDP-glucose/galactose) and ROS scavengers (ascorbate). Proteomic analysis (2-D electrophoresis and MALDI-TOF/TOF) revealed that the presence of alachlor led to upregulation of enzymes related to energy, sugar metabolism and ROS production. Further, overexpression of cyanide hydratase implicated the key role of this enzyme in the alachlor biodegradation pathway (Szewczyk et al., 2015).

The genomic analysis of *Burkholderia cenocepacia* CEIB S5-2 revealed the presence of key genes involved in glyphosate degradation pathways (sarcosine and AMPA pathway), suggesting that the bacterial strain could use both routes for glyphosate degradation. Genes *sox*A/B/D/G encoding sarcosine oxidase enzyme involved in sarcosine pathway as well as genes *gox* encoding glyphosate oxidoreductase, *mau*E/D encoding methylamine dehydrogenase and other genes encoding aminotransferases, phosphonatase enzymes involved in AMPA pathway were present on the genome (Diaz-Soto et al., 2024). Genomic data analysis of *Lysinibacillus sphaericus* suggested the presence of sarcosine oxidase gene and qRT-PCR analysis showed upregulation of this gene in presence of glyphosate (González-Valenzuela and Dussán, 2018).

Degradation pathway of chlorpyrifos and glyphosate in *Bacillus cereus* strains AKAD 3–1 were elucidated by GC-MS based metabolomics. Analysis of the intermediate and the final metabolic products confirmed that no toxic compounds were produced during chlorpyrifos and glyphosate degradation. This indicates that the bacterium harbors the metabolic pathway for detoxification and degradation of chlorpyrifos and glyphosate into non-toxic compounds (Malla et al., 2023).

Genomic analysis of *Sphingobacterium* sp. InxBP1 indicated the presence of various genes encoding enzymes involved in imidacloprid degradation. For example, nitronate monooxygenase (locus id K7A41_01745), amidohydrolase family enzymes or metal-dependent hydrolases (K7A41_03835, K7A41_07535) having similarity with 6-chloronicotinic acid chlorohydrolase, and FAD dependent monooxygenase (K7A41_12,275) similar to 6-hydroxy nicotinate monooxygenase, were found to be present in the genome, indicating the potential of strain InxBP1 to degrade imidacloprid (Gautam et al., 2023).

Therefore, omics techniques provide crucial data on various factors such as genes/proteins/metabolites involved, microbial community structure/dynamics, gene expression regulation and stress response mechanisms, thereby aiding in rational design of bioremediation and metabolic engineering strategies.

Application of natural isolates for bioremediation of CECs might face limitations such as slow degradation rates, incomplete transformation into toxic by-products, reduced survivability, and presence of simple carbon sources in the environment (Nielsen, 2001; Dvořák et al., 2017; Phale et al., 2020). These limitations can be overcome by directed genetic engineering approaches, called as “metabolic engineering”. These techniques can be used to broaden metabolic diversity, enhance degradation rates, enhance physiological vigour, overcome carbon catabolite repression *etc*. (Dvorak et al., 2017).

Multiple reports have described metabolic engineering of CEC degradation/transformation pathways in bacteria. For example, plasmid-mediated expression of sulfonamide monooxygenase and flavin reductase rendered *E. coli* BL21 (DE3) resistant to sulfamethoxazole (Kim D. et al., 2019). *E. coli* strain W, carrying the phenylacetic acid catabolic genes (*paa* operon) was engineered for penicillin utilisation by expression of *beta*-lactamase and penicillin amidase (*pga*) (Crofts et al., 2018). A consortium of *Aminobacter* sp. NyZ550 (that converts metformin to guanylurea) and metabolically engineered *P. putida* PaW340 (expressing guanylurea hydrolase GuuH) was constructed for metformin mineralisation. While strain NyZ550 converted metformin to guanylurea and dimethylamine, strain PaW340 metabolised guanylurea to guanidine, which was used as nitrogen source by strain NyZ550 (Li et al., 2023). Table 3 provides a comprehensive review of the application of metabolic engineering to CEC biodegradation.

**TABLE 3:** Metabolic engineering of CEC degradation pathways in various bacterial isolates.

Host organism	Target CEC	Mode of engineering	Overexpressed genes	Donor organism(s)	Reference
*Pseudomonas putida*	Chlorpyrifos	Plasmid-mediated overexpression	*ina*Q-*wlac*D (surface display anchor-fused laccase enzyme)	*Shigella dysenteriae*	Wang et al. (2012)
*Pseudomonas putida* KT2440	Chlorpyrifos	Suicide vector-mediated chromosomal integration	*mcd* (carbofuran hydrolase) *mpd* (chlorpyrifos hydrolase)	*Achromobacter* sp. strain WM111 *Stenotrophomonas* sp. YC-1	Gong et al. (2016)
*Escherichia coli* W	Penicillin	Plasmid-mediated overexpression	*bla* (*beta*-lactamase) *pga* (penicillin amidase)	- *Escherichia coli* W	Crofts et al. (2018)
*Escherichia coli* BL21 (DE3)	DEHP	Plasmid-mediated overexpression	*goest*15 (DEHP esterase) *goest*M1 (MEHP esterase)	*Gordonia* sp. 5F *Gordonia* sp. 5F	Huang et al. (2019)
*Escherichia coli* BL21 (DE3)	Sulfomethoxazole	Plasmid-mediated overexpression	*sul*X (sulfonamide monooxygenase) *sul*R (flavin reductase)	*Microbacterium* sp. CJ77	Kim et al. (2019a)
*Escherichia coli* JM109	Chlorpyrifos	Plasmid-mediated overexpression	*opd* (organophosphate hydrolase)	*Staphylococcus warner* *Pseudomonas putida* *Stenotrophomonas maltophilia*	John et al. (2020)
*Pseudomonas putida* PaW340	Guanylurea	Plasmid-mediated overexpression	*guu*H (guanylurea hydrolase)	*Pseudomonas mendocina*	Li et al. (2023)

## 6 Conclusion and future perspectives

Contaminants of emerging concern (CECs) are a heterogeneous group of naturally occurring or synthetic compounds that pose significant risk to human and ecological health due to their unregulated release into the environment. Among these, pharmaceuticals, cyanotoxins, plasticizers and pesticides have been found to occur in diverse habitats such as WWTPs, rivers, surface waters, soil as well as the atmosphere. Measures like precise monitoring of these compounds in various habitats, tracking their transport across ecological compartments and development of stringent regulatory policies might aid in mitigating risks at the point of release. Whereas, for already contaminated habitats, microbial remediation provides an eco-friendly and cost-effective solution. Microbes have adapted to these persistent compounds by the action of broad substrate specific enzymes (biotransformation) and evolution of metabolic pathways to utilise them as growth substrate, thereby mitigating the associated risks. The application of omics reveals various pathway components such as genes, transcripts, proteins, metabolites and their complex interactions, thereby facilitating development of efficient clean-up strategies. However, the available literature on CEC biodegradation primarily focuses on biotransformation products, while reports of complete mineralisation pathways and associated enzymes are limited. Such information is crucial for metabolic engineering applications and scaling-up the bioremediation process for efficient environmental clean-up, offering potential research opportunities.
